# TFPI2 Promotes Perivascular Migration in an Angiotropism Model of Melanoma

**DOI:** 10.3389/fonc.2021.662434

**Published:** 2021-06-24

**Authors:** Jing Mo, Xiulan Zhao, Wei Wang, Nan Zhao, Xueyi Dong, Yanhui Zhang, Runfen Cheng, Baocun Sun

**Affiliations:** ^1^ Department of Pathology, Tianjin Medical University, Tianjin, China; ^2^ Department of Pathology, General Hospital of Tianjin Medical University, Tianjin, China; ^3^ Department of Pathology, Cancer Hospital of Tianjin Medical University, Tianjin, China

**Keywords:** angiotropism, TFPI2, angiogenesis, metastasis, melanoma

## Abstract

**Purpose:**

Angiotropism is the process by which cancer cells attach to and migrate along blood vessels to acquire vasculature, disseminate, and metastasize. However, the molecular basis for such vessel–tumor interactions has not been fully elucidated, partly due to limited experimental models. In this study, we aimed to observe and explore the molecular mechanism underlying angiotropism in melanoma.

**Methods:**

To monitor the interactions of human melanoma cells with the vasculature *in vivo*, a murine coxenograft model was employed by co-injecting highly and poorly invasive melanoma cells subcutaneously. To identify key pathways and genes involved in the angiotropic phenotype of melanoma, analysis of differentially expressed genes (DEGs) and gene set enrichment analysis (GSEA) were performed. The role of tissue factor pathway inhibitor 2 (TFPI2) in angiotropism was evaluated by immunostaining, adhesion assay, shRNA, and *in vivo* tumorigenicity. Angiotropism and TFPI2 expression were examined in surgical specimens of melanoma by immunohistochemical staining. Data from The Cancer Genome Atlas (TCGA) were analyzed to explore the expression and prognostic implications of TFPI2 in uveal and cutaneous melanoma.

**Results:**

Highly invasive melanoma cells spread along the branches of intratumor blood vessels to the leading edge of invasion in the coxenograft model, resembling angiotropic migration. Mechanisms underlying angiotropism were primarily associated with molecular function regulators, regulation of cell population proliferation, developmental processes, cell differentiation, responses to cytokines and cell motility/locomotion. TFPI2 downregulation weakened the perivascular migration of highly invasive melanoma cells. High levels of TFPI2 were correlated with worse and better survival in uveal and cutaneous melanoma, respectively.

**Conclusion:**

These results provide a straightforward *in vivo* model for the observation of angiotropism and suggest that TFPI2 could inhibit the angiotropic phenotype of melanoma.

## Introduction

Metastasis, the main cause of death from cancer, is biologically a rather inefficient multistep process due to its complexity. Most cancer cells perish when leaving a solid tumor, and after intravasation, circulating tumor cells must survive, extravasate, and grow to form secondary tumors (1). While intravascular dissemination is now a well-accepted mechanism of spread, cancer cells can migrate along the blood vessels to further their growth and spread in multiple cancer types, including melanoma, lung cancer, hepatocellular carcinoma, glioma, and many metastatic diseases (2). The main hallmark of the above phenomenon is that cancer cells can attach to the abluminal surfaces of blood vessels (angiotropism and vessel co-option) and migrate along the external surface of vessels without any sign of intravasation (pericyte mimicry, PM) (3, 4). This biological process is similar to the migration of tumor cells along anatomic structures such as nerves and skin appendages, which has been previously recognized (5). This alternative travel route for metastasis, distinct from intravascular dissemination, has been termed extravascular migratory metastasis (EVMM), and angiotropism is the histological surrogate of EVMM (6). Given that angiotropism connotes both vessel co-option and continuous migration ability of tumor cells, we used the term angiotropism in the present study. Located in the vascular niches, cancer cells, which hijack both pre-existing and/or neoblood vessels, can survive and are resistant to various therapeutic approaches, particularly antiangiogenic treatment (7).

To effectively target angiotropism, it is essential to explore the molecular mechanisms that govern the angiotropic process. Studies of glioma showed that bradykinin, CXCR4, and Wnt7 were involved in glioma spread along vessels (8–10). Furthermore, adhesion molecules, metabolic signaling and pathways involving cancer cell motility were suggested to facilitate angiotropism or vessel co-option in various types of cancer (11, 12). Cancer cells also use contact guidance and chemotactic cues to reach their final metastatic sites (13). Angiotropism has been mainly studied and demonstrated as a prognostic factor predicting metastasis and survival in cutaneous melanoma (CM) and uveal melanoma (UM) (14–21). Angiotropic melanoma cells not only derive from the neural crest but also share the behaviors and mechanisms of migrating neural crest cells during embryogenesis (22). Molecular studies for angiotropism in melanoma have been previously reviewed (23). In particular, cell motility/migration, embryonic/cancer stem cells, inflammation, and pericyte markers are relevant to angiotropic melanoma.

Tissue factor pathway inhibitor 2 (TFPI-2) is a Kunitz-type serine protease inhibitor. By inhibiting a variety of proteinases, including plasmin, trypsin, and metalloproteinases (24–26), TFPI2 can suppress tumor invasion and metastasis by regulating extracellular matrix degradation. Downregulation of TFPI2 is present in various types of cancer and is associated with malignant tumor progression; moreover, methylation of TFPI2 can predict the prognosis and metastasis of malignancy (27–30). Based on these results, TFPI2 has been regarded as a tumor suppressor gene. However, TFPI2 has also been shown to promote the migration of tumor cells, support firm adhesion of endothelial cells, and enhance the formation of vasculogenic-like networks by aggressive melanoma cells (31–33). The mechanism underlying the diverse effects of TFPI2 remains largely unknown.

Multiple *in vivo* and *in vitro* models were used to study the process and mechanisms of angiotropism in different cancer types. In this study, we established a novel and effective angiotropic model of melanoma. Based on this model, we examined DEGs and pathways/biological processes involved in melanoma angiotropism. Furthermore, we found that TFPI2 could promote angiotropic migration of melanoma cells and that higher TFPI2 expression was correlated with worse and better survival of UM and CM patients, respectively, indicating its potentially diverse effects in a context-dependent manner.

## Materials And Methods

### Cell Culture

Melanoma cell lines (C918 and OCM-1A) and human umbilical vein endothelial cells (HUVECs) were obtained from the Cell Resource Center (Peking Union Medical College, China) and respectively cultured in Dulbecco’s Modified Eagle’s Medium (DMEM) and complete endothelial cell growth medium (ECG Medium; Provitro) supplemented with 10% fetal bovine serum (FBS, Invitrogen). The C918 cell line was derived from a cutaneous melanoma driven by KRAS, while the OCM-1A cell line was derived from a uveal melanoma driven by BRAF. The identity of the cell lines was verified by short tandem repeat (STR) analysis (Genewiz Inc.) in 2018.

### Lentivirus Production and Transduction

C918, OCM-1A, and HUVEC cells were infected with lentiviral particles expressing the green fluorescent protein (GFP), mCherry fluorescent protein (mCherry), and recombinant lentiviruses expressing shTFPI2 with a puromycin-resistance cassette in the corresponding experiments. Infection with GFP/mCherry viruses did not noticeably change the morphology and growth of cells. Human lentivirus GFP and mCherry expression plasmids and recombinant lentiviruses expressing shTFPI2 were constructed by GeneCopoeia Inc. (Guangzhou Science Park, Guangzhou, China). Transduction and cell selection were performed as described previously (34). Briefly, lentiviral particles were produced by cotransfection of 293T cells using a Lenti-Pac HIV Expression Packaging Kit (HPK-LvTR-40, GeneCopoeia Inc.) according to the manufacturer’s protocol. Supernatant containing viruses were collected 72 h post-transfection and filtered through a 0.45mm filter. For cell transduction, 5 × 10^4^ melanoma or HUVEC cells were seeded in six-well plates. A volume of 1 ml of the viral supernatant was added, and the volume of each well was adjusted to 2 ml with culture medium. Polybrene was added to a final concentration of 10 mg/ml and 10 h later the media was refreshed. Cells were selected with puromycin (2 mg/ml) for 2 weeks. Transduction efficiency was confirmed *via* fluorescence imaging and western blot analysis. TFPI2 shRNA target sequences are #1GCCTTTATGGTTGTATCTGAA; #2 CCTGTGATGCTTTCACCTATA; #3 GAACAGGTTTCCAGATGAAGC. Control non-target shRNA: CAACAAGATGAAGAGCACCAA. The No.1 shRNA-TFPI2 sequence was chosen for subsequent studies.

### Cell Invasion Assay

Melanoma cells were starved overnight in FBS-free DMEM medium. Cell invasion assay was performed using Transwell chambers (8 μm pore size, BD Biosciences, CA, USA). Briefly, each Transwell membrane is covered by 100 μl thawed Matrigel (BD Biosciences) diluted 1:4 with DMED. The 2 × 10^4^ melanoma cells in 200 μl FBS-free DMEM medium were inoculated into the upper chamber, and 10% FBS as chemoattractant was added to the lower compartment. After incubation for 24 h and Matrigel removal, the filter was stained with 0.1% crystal violet in distilled water (w/v). The invasiveness was determined by counting the penetrated cells under a microscope of three random fields in each well.

### Cell Migration Assay

Pipette tip scratch assay was used to determine migration ability of melanoma cell lines. Melanoma cell suspension (400,000 cells) was seeded into each well on six-well plates, allowing cells to adhere and spread until they create a confluent monolayer. Cells were scratched using a 200 µl pipette tip, then washed with PBS and media was refreshed. Images of the wounds were collected at 0, 24, and 48h after scratching. Cell motility was assessed by analyzing the distance of the cells from each side of the wound using ImageJ. Each experiment was performed in triplicate.

### Cell–Cell Adhesion Assay

Cell–cell adhesion assay was conducted using melanoma-GFP and HUVECs-mCherry cells under basic conditions as previously described (35). Briefly, 100,000 HUVEC cells per well were seeded on 24-well plates and allowed to adhere overnight. Then, 100,000 melanoma cells were added and co-incubated with HUVECs for 40 min at 37°C, 5% CO_2_. Unattached cells were washed off with PBS, and fluorescent images were captured subsequently. Triplicates were used for each condition.

### *In Vivo* Tumorigenicity

Four-week-old male BALB/c-nu/nu mice (Beijing HFK Bioscience Co. Ltd, Beijing, China) were subcutaneously injected with 5 × 10^6^ C918-GFP cells (stably transfected with GFP) alone, OCM-1A-con cells alone, a mixture of equal amounts of C918-GFP cells with OCM-1A-con cells, and a mixture of equal amounts of C918-sh-TFPI2 cells (stably transfected with sh-TFPI2-GFP) with OCM-1A-sh-con cells per mouse. Tumor formation/growth was monitored weekly using calipers, and tumor volumes (TVs) were determined using the formula [TV (mm^3^) = π/6 × 0.5 × length × (width)^2^] (36). Mice with the tumor exceeding 10 mm diameter were sacrificed, and xenografts were harvested and fixed with phosphate-buffered neutral formalin and prepared for standard histological examination. All animal experiments were carried out in agreement with ethical regulations and protocols approved by the Institutional Animal Care and Use Committee of Tianjin Medical University.

### Immunohistochemistry and Endomucin-PAS (Periodic Acid Schiff) Double Staining

Sections 4 μm in thickness were de-paraffinized and rehydrated by standard protocols. Antigen retrieval was performed by heating slides in retrieval buffers pH6 in a microwave. Sections were incubated in blocking buffer (goat serum) for 1 h at room temperature and followed by incubation overnight at 4°C with primary antibodies against Ki-67 (ZSGB-Bio, ZM-0166), GFP (Abcam, ab6556, dilution 1:500), TFPI2 (Abcam, ab186747, dilution 1:100), and endomucin (Ebioscience, 14-5851, dilution 1:1,000). Sections were incubated with the secondary antibodies (rabbit (PV-6001), mouse (PV-6002), and rat (PV-6004) polymer detection system, ZSGB-Bio) for 1 h at room temperature and then stained with DAB and counterstained with hematoxylin. Staining intensity scoring was performed as previously described (37). Endomucin-PAS staining was performed after staining of endomucin with DAB. Sections were incubated with 0.5% periodic acid for 15 min, rinsed in distilled water for 10 min, Schiff’s reagent for 30 min, and counterstained with hematoxylin (38).

### Western Blotting

Cell supernatant or lysates derived from C918 and OCM-1A cells were used to examine TFPI2 protein levels. For cell supernatant collection, media of culturing cells were refreshed with FBS-free DMEM. Supernatant and cell lysates were obtained 48 h later. Supernatant enrichment was performed using a 10 kDa ultrafiltration tube (50 ml, Millipore) and centrifuged. Western blotting was done as described previously (38). Whole cell lysate was separated, transferred onto PVDF membranes, and probed with TFPI2 (Abcam, ab186747, 1:500) and GAPDH (Santa Cruz Biotechnology, sc25778, 1:2,000). Blots of target proteins were detected with WesternBright ECL HRP substrate (R-03031-D2, advansta) and visualized using a C-DiGit Blot Scanner (LI-COR Biosciences).

### Bioinformatics Analyses and Pathway Gene Signatures Analyzed Using GSEA

Gene enrichment analysis was performed using STRING web platform (https://string-db.org/). GSEA was performed using the GSEA software (version 4.0.3) (https://www.gsea-msigdb.org/gsea/index.jsp). GSEA is a computational method for exploring whether a given gene set is significantly enriched in a group of gene markers ranked by their relevance with a phenotype of interest. The gene ontology (GO) gene set dataset was used to compare the pathways in highly and poorly invasive melanoma cell lines. The upregulated pathways were defined by a normalized enrichment score (NES) >0 and the downregulated pathways by NES <0. Pathways with a false discovery rate *P* ≤0.1 were considered significantly enriched. The Venn diagram was produced using InteractiVenn (http://bioinformatics.psb.ugent.be/webtools/Venn/).

### Datasets

Transcriptome profiling and prognostic data of CM and UM were accessed from the TCGA website (cancergenome.nih.gov). Among them, metastasis (+) *vs.* metastasis (−) (249 *vs.* 219 in CM and 27 *vs.* 53 in UM) were available based on clinical diagnosis. To screen out DEGs between the metastasis (+) and metastasis (−) groups, the R package ‘edgeR’ was applied in R Studio. A fold change >1 and *P <*0.05 were considered to indicate a statistically significant difference. DEGs between highly and poorly invasive melanoma cells were obtained from microarray analysis by Hendrix and Folberg (referred to as DEGs of cell lines) (39, 40).

### Patient Tissue Samples

This study included 135 primary human CM from patients who underwent surgical resection in Cancer Institute and Hospital of Tianjin Medical University in China from January 1996 to December 2014 (referred as TMU-CM cohort). All human studies have been examined by the Ethics Committee of Tianjin Medical University.

### Statistical Analysis

Statistical analyses were conducted using SPSS version 17.0 software (SPSS Inc., Chicago, IL, USA) and GraphPad Prism6 (GraphPad Sofware, Inc., San Diego, CA, USA). To compare the continuous variables, differences between two groups were analyzed using the Student’s *t*-test or Mann–Whitney *U*-test. To compare the categorical variables, chi-square test was performed to assess the pathological and clinical characteristics of the TFPI2 positive/negative groups and angiotropism present/absent groups. Optimal cut-off points were determined as described previously (34). Survival time between groups was evaluated using Kaplan–Meier method and compared by log-rank test. Univariate and multivariate analyses were performed by Cox proportional hazards models. Statistical significance was defined as *P* < 0.05.

## Results

### Angiotropism Model Using Highly and Poorly Invasive Melanoma Cell Lines

**Figure 1:**
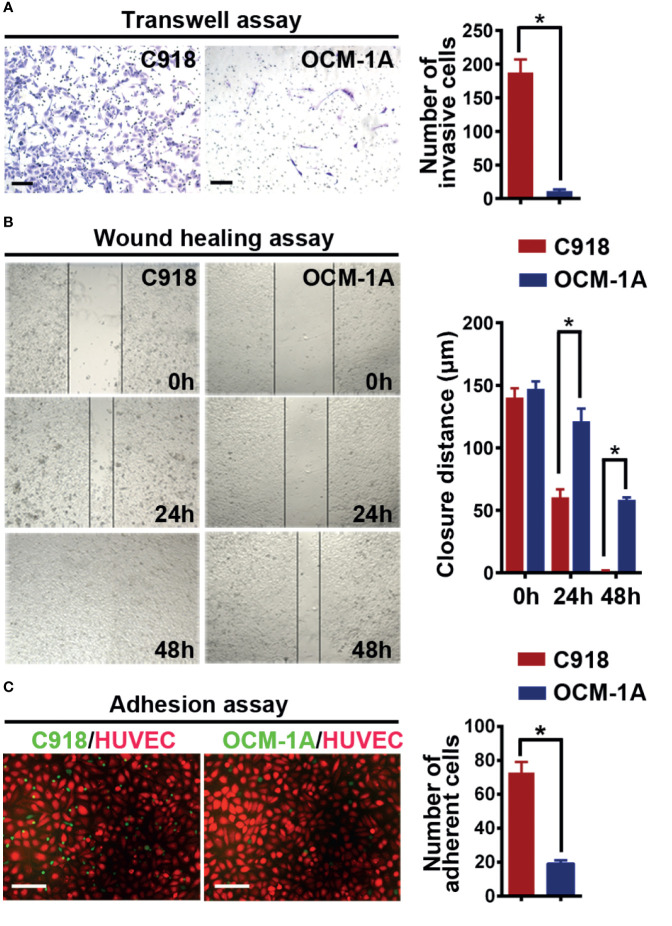
C918 cells show higher invasion/migration ability and stronger interaction with the endothelium. **(A)** Transwell invasion assay reveals higher invasive ability of C918 cells when compared with OCM-1A cells. The average numbers of invasive C918 or OCM-1A cells per three non-overlapping fields were counted (minimum of three trials each) (*t* test; *P* < 0.0001). **(B)** Wound healing assay reveals faster migration of C918 cells when compared with OCM-1A cells. The closure rate of cells in the wound healing assay was determined for C918 and OCM-1A cell lines (minimum of three trials each) (*t* test; 0 h, *P* = 0.22, 24 h, *P* = 0.0054; 48 h, *P* = 0.0004). **(C)** Numbers of adherent melanoma cells (green) on HUVECs (red) are shown. Melanoma-GFP cells remaining on the monolayer of HUVEC-mCherry cells were counted (10 fields from three independent experiments) (*P* < 0.0001). Scale bar 100 μm. *P < 0.05.

Angiotropism involves the migration ability of cancer cells along blood vessels. It is reasonable to infer that cancer cell motility may be required for angiotropism. Therefore, to validate the different invasive abilities of the C918 and OCM-1A cells used in this study, invasion and migration assays were performed. The results showed a higher invasion and migration ability of C918 cells than OCM-1A cells (**Figures 1A, B** **)**, consistent with findings reported by Mary Hendrix (40).

To verify whether the C918 cell line has a greater ability than OCM-1A cells to interact with endothelium *in vitro*, we next cocultured melanoma cells with endothelial cells or with aortic rings *in vitro* (41). Melanoma-mCherry cells were cocultured with HUVEC-GFP cells, and melanoma-GFP cells were cocultured with aortic rings. The results showed that more C918 cells migrated towards and along endothelial cell surfaces and aortic rings than OCM-1A cells (**Supplementary Figures 1A, B** **)**. Further evidence for endothelial–melanoma cell interactions was obtained by cell–cell adhesion assays. Similar results were obtained in which more C918 cells adhered to the surface of the HUVEC cell monolayer compared to OCM-1A cells under static conditions (**Figure 1C**).

**Figure 2:**
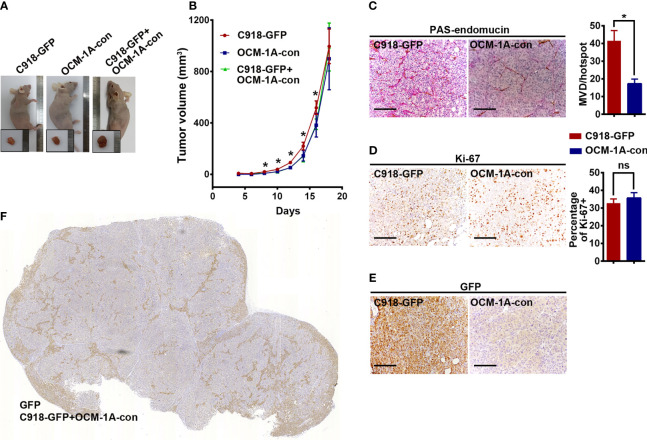
No difference in tumorigenesis derived from xenografts alone or coxenografts. **(A)** Representative mice and corresponding tumors 18 days after subcutaneous injection of C918-GFP, OCM-1A-con and C918-GFP + OCM-1A-con cells *P < 0.05. **(B)** The statistics of tumor volume at different times after subcutaneous injection are shown (n = 6). **(C–E)** Representative images, stained for PAS-endomucin to identify blood vessels (*P* < 0.0001), Ki-67 to evaluate cell proliferation (*P* = 0.12) and GFP to verify lentiviral infection efficiency in xenografts of C918-GFP and OCM-1A-con cells. Scale bar 100 μm. **(F)** Optical scans stained for GFP in coxenograft tissue sections (C918-GFP+OCM-1A-con) showing the distribution of C918-GFP cells. Scale bar 1 mm; ns, not significant.

To investigate the superiority of the highly invasive capacity of C918 over poorly invasive OCM-1A cells in coxenografts, a mixture of C918-GFP and OCM-1A-con cells (coxenograft), C918-GFP cells alone or OCM-1A-con cells alone was subcutaneously injected into the flanks of recipient BALB/c nude mice. Palpable tumors formed in all of these groups approximately 10 days after injection. There were no significant differences in tumorigenicity or rate of tumor growth between these groups, likely due to little difference in proliferative capacity and microvessel density (MVD) between these cell lines (**Figures 2A–D**).

**Figure 3:**
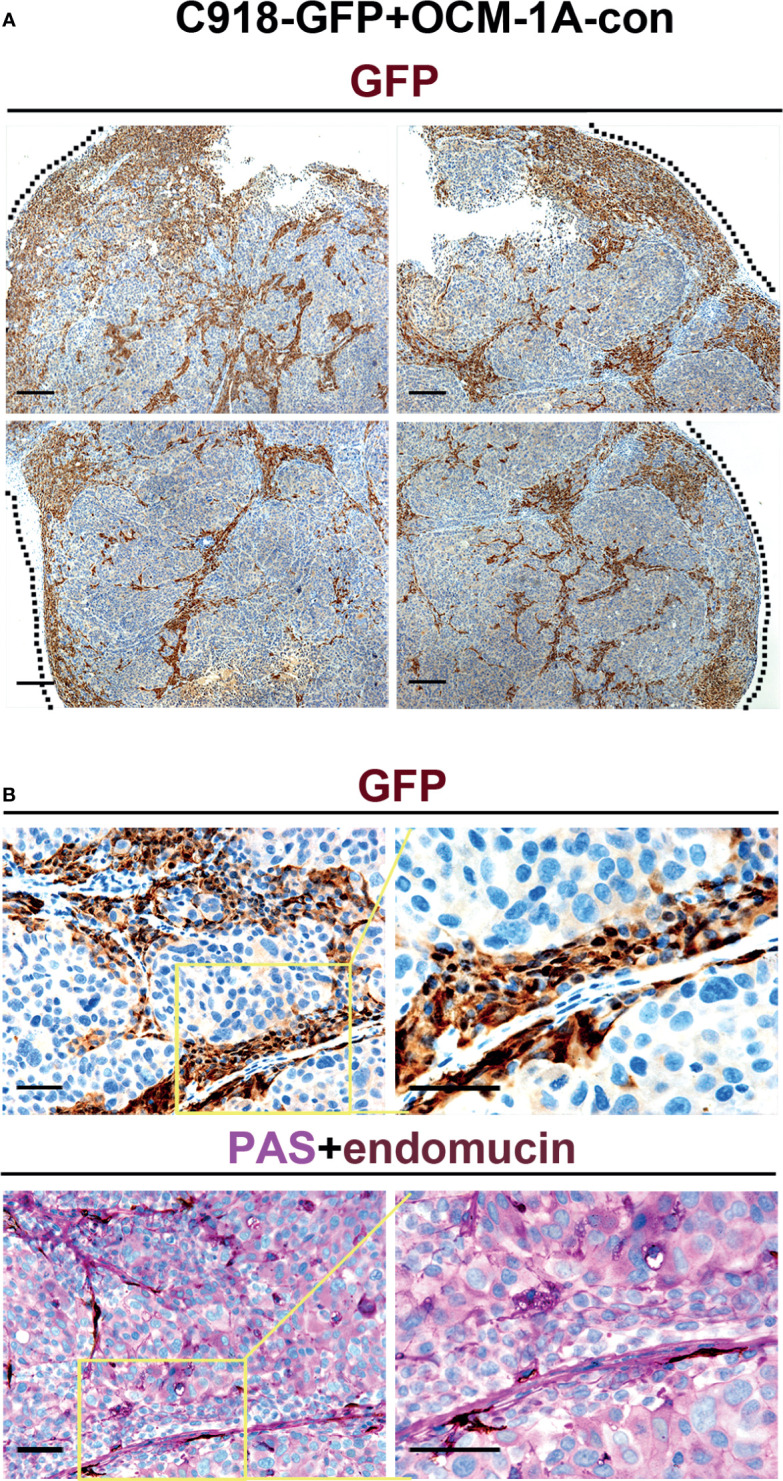
Angiotropism model generated by coxenografting highly and poorly invasive melanoma cells. **(A)** C918-GFP and OCM-1A-con cells were coinjected subcutaneously into BALB/c-nu/nu mice. By staining for GFP, C918-GFP cells (dark brown) exhibited a conspicuous pericytic location along the blood vessels compared with OCM-1A-con cells. Note the progression of C918-GFP cells along vessels and extending into the tumor invasive front (dashed line) in the low magnifications of four directions of a coxenograft. Scale bar 100 μm. **(B)** Representative GFP-stained sections show the localization of C918-GFP cells on the external surfaces of blood vessels (upper panel). Evidence of intravascular melanoma cells was not identified. Corresponding serial sections were stained for PAS-endomucin. Blood vessels were positively stained by endomucin throughout the tumors and correlated with the characteristic extracellular matrix-rich patterns (lower panel). Scale bar 50 μm.

Evaluation of GFP expression indicated that almost all C918 cells, with different degrees of staining intensity, were GFP-positive in C918-GFP xenografts, while OCM-1A cells were GFP-negative in OCM-1A-con xenografts (**Figures 2E, F** and **Supplementary Figure 2**). This result ensures the credibility that GFP-positive cells could be used as markers for tracing C918 cells. In the coxenograft (**Figure 3**), a striking spread of GFP-labeled C918 cells along the trunk and branches of intratumor blood vessels was observed by GFP and PAS-endomucin staining. In contrast, OCM-1A cells tended to grow as tumor aggregates supplied by circumjacent and ingrowing microvasculature. By scanning the entire tissue section of the coxenograft, we found that more C918 than OCM-1A cells extended along blood vessels to the advancing front or periphery of the tumor (**Figure 2F** and **Supplementary Figure 2**). Such histological morphology resembled two biological phenomena, angiotropism and blood vessel co-option, both of which indicate the organization of tumor cells cuffing around pre-existing or newly formed blood vessels. The above data show that it is practicable and valuable to use coxenografts of melanoma cell lines, which have distinctly different invasive and angiotropic abilities, as an alternative laboratory model of angiotropism.

### Gene Signature of Highly and Poorly Invasive Melanoma Cell Lines

DEGs between C918 and OCM-1A cell lines were obtained from the study by Mary Hendrix. A total of 75 genes were found to be differentially expressed more than 4.6-fold, of which 49 genes were differentially upregulated and 26 were downregulated in the C918 cell line (**Supplementary Table 1**). These DEGs were confirmed again in Folberg R’s study by comparing the same two cell lines (39). Next, to analyze the biological processes and mechanisms related to DEGs in this microarray, GSEA was performed based on the predefined gene sets in the Molecular Signatures Database (http://www.broadinstitute.org/gsea/msigdb/index.jsp) (42). Eighteen gene sets upregulated in the C918 cell line were identified by GSEA, while no gene sets were enriched in OCM-1A cells. After selecting gene sets with NES >1.0, the top seven enrichment plots of the gene set/Gene Ontology (GO) terms were enriched, including molecular function regulator, regulation of cell population proliferation, cell motility, positive regulation of developmental process, regulation of cell differentiation, response to cytokine, and locomotion (**Supplementary Figure 3A**). We next used leading-edge analysis to extract the core genes in the top seven gene sets, which revealed that 20 genes, BNC1, TGM1, PLAU, JUN, LIF, VEGFC, MME, HCLS1, KRT8, TRIM22, ARHGDIB, TFPI2, TFPI, SAA1, BIRC3, SEMA4D, FBN2, PAX8, PKP2, and FBN1, contributed most to the enrichment score and were regarded as drivers of enrichment (**Supplementary Figure 3B**). To confirm the biological function of these core genes using an alternative platform, the 20 core genes were uploaded to and analyzed by STRING. Similar results were obtained when analysis of enriched molecular functions, biological processes, and KEGG pathways was performed (**Supplementary Figure 4**). Taken together, these results revealed key pathways and genes associated with highly and poorly invasive melanoma cell lines in the coxenograft model.

Additionally, to evaluate the impact of the expression levels of the 20 genes on the overall survival of melanoma patients, 80 UM and 435 CM patients with complete RNA sequencing and clinical data from TCGA-UM and TCGA-CM were used. In UM, univariate Cox regression analyses indicated that LIF, VEGFC, and TFPI2 were significantly associated with survival time, and by multivariate Cox regression analyses, none of these three genes served as an independent risk factor for UM patient prognosis (**Supplementary Table 2**). In CM, LIF, HCLS1, TRIM22, ARHGDIB, BIRC3, and SEMA4D were significantly associated with survival time in univariate Cox regression analyses, and TRIM22 and SEMA4D served as independent risk factors for CM patient prognosis (**Supplementary Table 3**).

### High Expression of TFPI2 in Highly Invasive Cells and Knockdown of TFPI2 Inhibits Perivascular Migration

**Figure 4:**
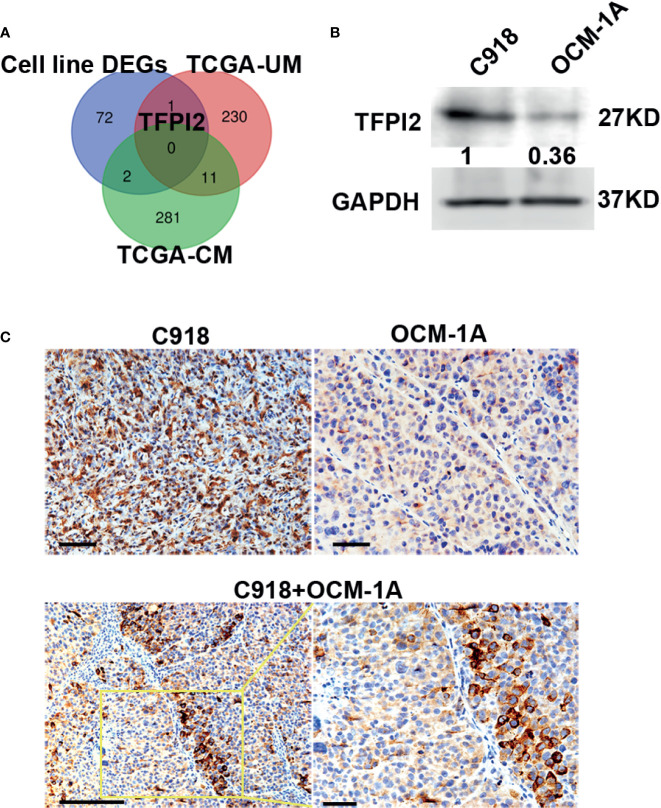
High levels of TFPI2 in the highly invasive melanoma cell line. **(A)** A Venn diagram shows overlaps of DEGs from three datasets. TFPI2 was consistently upregulated in the C918 cell line and metastasis (+) UM. **(B)** Western blotting shows higher levels of TFPI2 in C918 cells than in OCM-1A cells in whole cell lysate. **(C)** Representative IHC images stained for TFPI2 showing strikingly strong and weak expression of TFPI2 in xenografts of C918 and OCM-1A cells, respectively (upper panel). TFPI2-positive melanoma cells predominately disposed along the vascular channels in the coxenograft (C918 + OCM-1A) (lower panel). Scale bar 50 μm.

Because highly invasive C918 cells also displayed strong metastatic potential (40), to address whether genes involved in different characteristics of these two melanoma cell lines could also play a role in the process of metastasis, we first sought to identify genes that correlate with melanoma metastasis using gene expression profiles of melanoma cases with metastasis (+) and without metastasis (−) extracted from TCGA. Gene transcripts with log2FC >1 or log2FC <−1 and Benjamini–Hochberg adj. *P* < 0.05 were considered to be DEGs. Compared with the metastasis (−) group, we identified 242 DEGs in UM, of which 61 were significantly increased in metastasis (+) and 294 DEGs in CM, of which 32 were significantly increased in metastasis (+). Then, a Venn diagram was generated to visualize the overlapping DEGs in the cell line DEGs (Hendrix’ study), TCGA-UM, and TCGA-CM datasets. No gene overlap among these three datasets was identified. Between cell line DEGs and TCGA-UM datasets, one overlapping gene, TFPI2, also the core gene in our leading-edge analysis, was identified (**Figure 4A**).

TFPI2 inhibits extracellular matrix (ECM) degradation, and low TFPI2 levels are associated with disease progression (43–46). To confirm the expression of TFPI2, we detected TFPI2 using western blot and IHC staining. TFPI2 expression was significantly higher in C918 cells than in OCM-1A cells both in whole cell lysate and supernatants of cell cultures (**Figure 4B** and **Supplementary Figure 5**). Moreover, staining for TFPI2 was clearly detected in C918 xenografts and prominently located at areas adjacent to capillaries in coxenografts. However, this was not the case for OCM-1A xenografts and areas far away from the blood vessels of coxenografts (**Figure 4C**).

**Figure 5:**
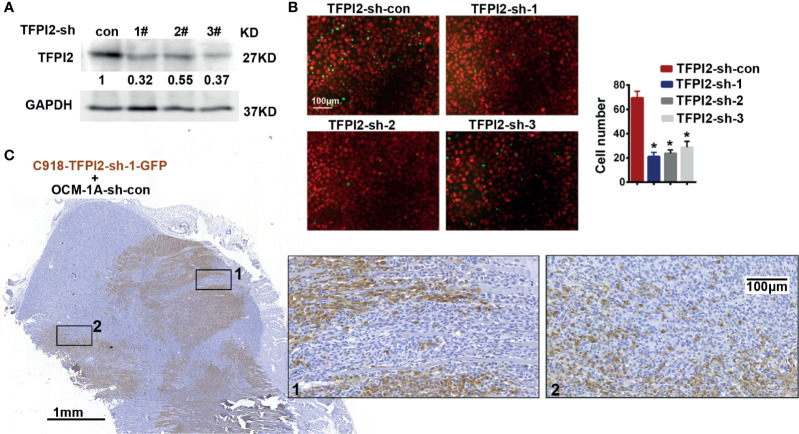
Knockdown of TFPI2 inhibits angiotropic migration of the highly invasive cell line. **(A)** TFPI2 expression levels in whole cell lysate were determined by western blotting and quantitated relative to sh-con cells. **(B)** Number of adherent C918 cells with normal TFPI2 levels (sh-con) or TFPI2 knockdown (sh-TFPI2-1-3) are shown and counted. *P < 0.05. **(C)** The distribution of C918 cells with TFPI2 knockdown is shown in a coxenograft model. GFP staining is shown. Dark brown represents C918-sh-TFPI2-GFP cells, and GFP-negative areas indicate OCM-1A-sh-con cells.

To address whether TFPI2 could have a functional role in the process of perivascular migration *in vivo*, we used the abovementioned coxenograft model. We first attempted to knock down TFPI2 expression in C918 cells using three different TFPI2-targeted lentiviruses. One of these sh-TFPI2s (sh-TFPI2-1) significantly reduced TFPI2 expression (**Figure 5A**, **Supplementary Figure 5**). TFPI2 knockdown significantly suppressed the adhesion of C918 cells to HUVECs (**Figure 5B**). *In vivo*, C918-sh-TFPI2 (GFP-labeled) cells were subcutaneously coinjected with OCM-1A-sh-con cells. Notably, TFPI2 knockdown decreased the migration of C918 cells along blood vessels, reducing the presence of GFP-positive C918 cells at the periphery of the tumor (**Figure 5C**). These data indicate that the suppression of TFPI2 could block the ability of highly invasive melanoma cells to migrate along vessels *in vivo*.

### High Levels of TFPI2 Indicate Worse Survival of TCGA-UM Patients

**Figure 6:**
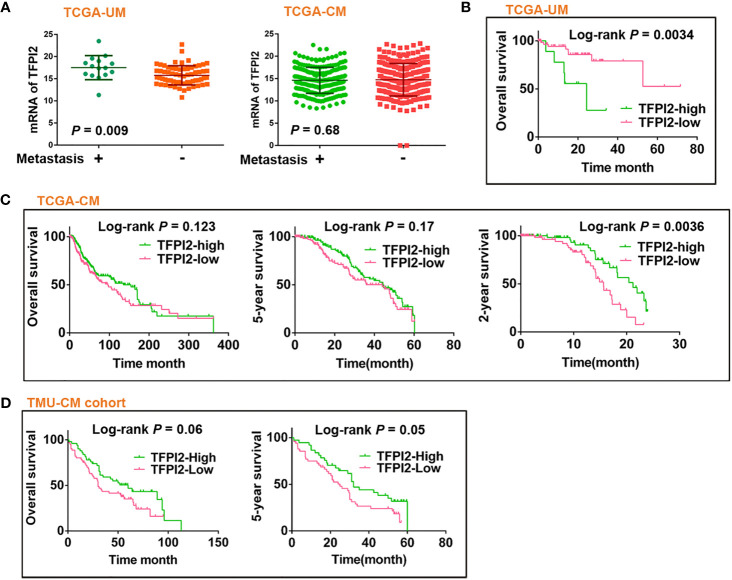
Prognostic significance of TFPI2 in melanoma patients. **(A)** Dot plot represents mRNA levels of TFPI2 in metastasis (+) *vs.* mestastasis (−) groups of TCGA-UM and TCGA-CM data, respectively. **(B–D)** Kaplan–Meier estimates of survival time for TCGA-UM, TCGA-CM, and TMU-CM cohort patients who were stratified into two groups: high and low levels of TFPI2.

The levels of TFPI2 were higher in the metastasis (+) group than in the metastasis (−) group of UM, but there was no difference between the metastasis (+) and metastasis (−) groups of CM (**Figure 6A**). Then, to assess the role of TFPI2 in clinical prognosis, Kaplan–Meier estimates of overall survival (OS) were calculated for TCGA-UM individuals. Patients were stratified into groups of high and low levels of TFPI2 based on the optimal cut-off value of TFPI2 mRNA (cut-off = 14.19; range 0–22.87). Notably, individuals with low levels of TFPI2 expression had a significantly better OS than patients with high levels of TFPI2 expression (*P* = 0.0034) (**Figure 6B**).

Using the same data set, univariate and multivariate Cox regression analyses were used to analyze the impact of age, sex, tumor thickness, diameter, mitotic count, stage, metastasis, and TFPI2 expression on TCGA-UM patient overall survival. Univariate analysis indicated that age, stage, TFPI2, and metastasis, but not tumor thickness, mitotic count or diameter, were significantly associated with overall survival in the UM patients (*P* = 0.02 for age of diagnosis, 0.008 for stage and metastasis, 0.003 for TFPI2). Based on the optimal cut-off value of TFPI2, its HR was 1.4 (95% confidence interval (CI): 1.1–1.7). In multivariate analyses, the TFPI2 effect did not remain significant after adjusting for age (*P* = 0.001, HR 1.108, 95% CI: 1.04–1.18), stage (*P* = 0.021, HR 4.27, 95% CI: 1.24–14.7) or metastasis (*P* = 0.01, HR = 8.36, 95% CI: 1.66–42.1) (**Supplementary Table 4**). We also examined the correlation between TFPI2 and baseline characteristics of melanoma patients from TCGA-UM. TFPI2 showed significant correlations with tumor diameter (*P* = 0.027), mitotic count (*P* = 0.016), stage (*P* = 0.0045) and metastasis (*P* = 0.0019) (**Supplementary Table 5**). These results indicate that evaluating the expression of TFPI2 in UM might help clarify which patients are at risk for disease progression.

### Potentially Discrepant Role of TFPI2 in UM and CM

In contrast to the observations in the TCGA-UM patient cohort, TCGA-CM and TMU-CM cohort patients with high TFPI2 expression showed significantly better 2- and 5-year survival, respectively, than patients with low TFPI2 expression (**Figures 6C, D** **)**. Hence, we investigated whether TFPI2 levels were correlated with the clinical characteristics of CM patients. The TFPI2 level in TCGA-CM was only significantly correlated with the age at diagnosis (*P* = 0.0064), while the R-squared value was low (−0.13) (**Supplementary Table 6**). By subjecting the TFPI2 level in the TCGA-CM cohort to univariate Cox proportional hazard regression analysis, TFPI2 did not correlate with the OS of CM patients compared to UM patients (**Supplementary Table 7**). Subsequently, the correlation between TFPI2 and TMU-CM cohort clinicopathological factors was analyzed. Although age, sex, ulceration, stage, LN metastasis, and remote metastasis did not show statistically significant correlations with TFPI2, there seemed to be a trend towards significance between the positive staining of TFPI2 with advanced stages (*P* = 0.09) and lymph node metastasis (*P* = 0.07) in the TMU-CM cohort (**Supplementary Table 8**). These results may imply different, potentially opposite, roles of TFPI2 in the survival of UM and CM patients.

To interpret the potentially discrepant role of TFPI2 in UM and CM, mRNA expression and methylation levels of TFPI2 in two types of melanoma in the TCGA data sets were analyzed. Both TFPI2 mRNA levels (*P* = 0.0003) and the frequency of TFPI2 methylation (*P* < 0.0001) were significantly higher in UM than in CM (**Supplementary Figure 6**). We next analyzed the coexpressed genes of TFPI2 in UM and CM using cBioPortal online tools (47, 48). Using the cut-off value of Spearman’s correlation coefficient of 0.4 and adjusted *P <*0.005, a total of 1,428 positively correlated and 604 negatively correlated genes exhibited significant coexpression with TFPI2 in UM. In CM patients, a total of 22 genes (eighteen positively correlated and four negatively correlated) showed significant coexpression with TFPI2 (**Supplementary Material Data Sheets 1** and **2**). Eleven of the coexpressed genes were shared by UM and CM. STRING and GSEA were used to analyze the potential mechanisms of the coexpressed genes, and the main KEGG pathways and top GO terms obtained are shown in **Supplementary Figure 7**. These findings suggest that most coexpressed genes and corresponding pathways are unique for UM and CM.

### Angiotropism Correlates With TFPI2 Expression

**Figure 7:**
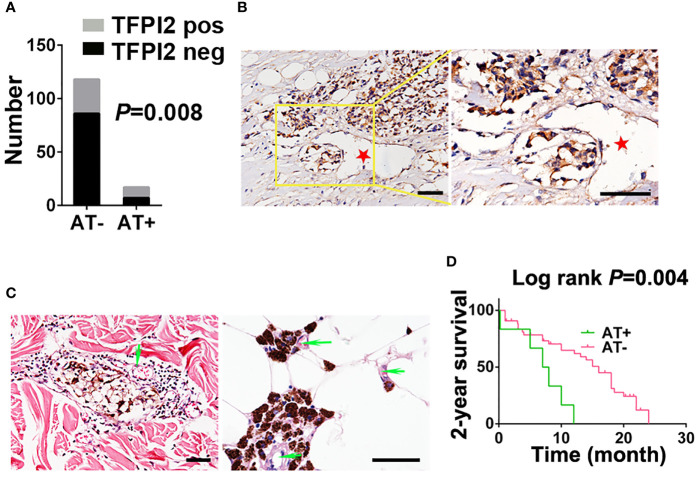
Correlation between TFPI2 expression and angiotropism and prognostic significance of angiotropism in melanoma patients. **(A)** The histogram shows the proportions of TFPI2-positive staining in the angiotropism-present (AT+) and angiotropism-absent (AT−) groups of the TMU-CM cohort. **(B)** Representative IHC staining images for TFPI2 show that melanoma cells cuffing the external surfaces of blood vessels (stars) were TFPI2-positive. Scale bar 50 μm. **(C)** Representative HE images show that pigmented melanoma cells were closely apposed to the external surface of blood vessels (arrows) in the dermis (left) and adipose tissue (right). **(D)** Kaplan–Meier estimates of 2-year survival for TMU-CM cohort patients who were stratified into two groups: angiotropism-present and angiotropism-absent.

We next examined whether TFPI2 is correlated with melanoma angiotropism by examining TFPI2 expression and distribution and its correlation with the presence of angiotropism in the TMU-CM cohort. By IHC staining, TFPI2-positive tissue sections were detected in 58.8% (10 of 17) of the angiotropism-present group and 27.1% (32 of 118) of the angiotropism-absent group (*P* = 0.008) (**Figure 7A**). The histopathological distribution of TFPI2-positive melanoma cells coopting tumor vessels is presented in **Figure 7B**. These data show that TFPI2 expression predominates in melanoma in the presence of angiotropism.

Because angiotropism is closely related to the metastasis and prognosis of various tumors (49), we retrospectively investigated the TMU-CM cohort to study the prognostic value of angiotropism in predicting the outcome of CM patients. Angiotropism was defined as melanoma cells closely apposed to vascular channels at the advancing front or in nearby tissue without intravasation (**Figure 7C**). Of the 135 cases, we observed typical angiotropism in 17 cases according to the above criteria. Angiotropism-present patients exhibited shorter survival durations than angiotropism-absent patients in the 2-year survival analysis (*P* = 0.004) (**Figure 7D**) but not in the 5-year (*P* = 0.59) or overall survival analysis (*P* = 0.3) (**Supplementary Figure 8**). We further investigated the correlation between age, sex, ulceration, stage, lymph node metastasis and metastasis with the presence of angiotropism, and no significant results were obtained (**Supplementary Table 9**). The lack of association is possibly due to the relatively small tumor cohort, and a larger cohort will be required to validate these findings.

## Discussion

Angiotropism (vessel co-option, pericytic mimicry, or extravascular migration) has been described and is believed to represent alternative means of melanoma dissemination and resistance to antitumor therapy (50). In the present study, we showed that (i) angiotropism could be simulated by coxenografting highly and poorly invasive melanoma cell lines *in vivo*; (ii) the role of TFPI2 in promoting perivascular migration, as well as key genes and signaling pathways involved in the angiotropic phenotype, was identified.

### Coxenograft Model of Angiotropism

To study the interaction between human melanoma cells and blood vessels, we used a murine model by inoculating a mixture of highly and poorly invasive melanoma cell lines subcutaneously. This relatively avascular microenvironment induces angiogenesis in xenografts but does not incorporate host vessels. It is important to note that angiogenesis involves newly formed vessels from preexisting vessels into the tumor periphery and center; next, after a sufficiently long period of observation, co-option of host vessels may be detected as an alternative blood supply. In this model, highly and poorly invasive melanoma cells were cotransplanted subcutaneously, allowing direct comparison of angiotropic ability and observation of angiogenesis involvement. There are still three main questions about this model.

First: Are melanoma cells cuffing microvessels in the coxenograft actually angiotropic? According to the definition, angiotropic tumor cells are histologically located at the advancing front or periphery or in nearby uninvolved tissue of the tumor (51). Our observation appears not to conform to the criteria. To think differently, if the poorly invasive cells were regarded as a “non-tumor tissue” component, it is reasonable to confirm the angiotropic attribute of highly invasive cells described in the present study.

Second: Did the new vessels induced by melanoma cells sprout towards C918 cells or did C918 spread and grow closely apposed to the abluminal surfaces of vessels? Poorly invasive OCM-1A cells in the coxenograft, equally supplied with angiogenic blood, grew as aggregates or nests and were mainly localized in the tumor center. Comparing the growth pattern and expansive distribution of C918 with OCM-1A, it is reasonable to believe that C918 indeed migrated along new vessels through the tumor mass and to the invasive front. Moreover, distinct and high levels of proangiogenic factors were detected in C918 cell lines (unpublished data), indicating a stronger effect of endothelium recruitment by C918. In particular, the cytokine interleukin-6 (IL-6), which is secreted by C918 but not present in OCM-1A, plays diverse and important roles in cancer cell migration, cancer progression, and epithelial-to-mesenchymal transition and can be triggered after human melanoma cell–endothelial interactions (52).

Third: Are C918 (deprecated name: MUM-2B) and OCM-1A (deprecated name: MUM-2C) cell lines cloned from a heterogeneous liver metastasis from one patient eye? C918 and OCM-1A were once regarded to be cloned from a heterogeneous liver metastasis from one patient eye (40). However, STR profiles for these two cell lines (obtained from original stock sources) indicated that OCM-1A and MUM-2C, being of uveal origin with a BRAF (V600E) mutation, were from the same patient and that M619, C918, and MUM-2B were from the same patient (53, 54). In addition, the C918 (MUM-2B) cell line has an identical STR profile to the cutaneous melanoma C8161 cell line driven by KRAS (55). These two melanoma cell lines used in this study are not syngeneic, and they are not typical cell lines of uveal or cutaneous melanoma due to genetic mutations. Even so, these two melanoma cell lines did show distinct invasive abilities, which was satisfactory for our study.

This model may not be optimal, but to the best of our knowledge, this coxenograft model is the first attempt to mix two cancer cell lines to directly observe angiotropism. This coxenograft model may represent a new and interesting approach to observe relationships between angiotropic tumor cells and co-opted blood channels. Additional studies using other types of cancer cells are needed to provide further evidence and verify the validity of this model.

### Molecular Mechanisms of Angiotropism

The interaction between angiotropic cancer cells and blood vessels is a complicated biochemical process that may involve both proangiogenic factors and tumor-intrinsic signaling factors. Although Hendrix et al. revealed a variety of DEGs expressed by highly and poorly invasive melanoma cell lines in their study; they did not perform further analysis.

Based on cell line DEGs and public data, we explored the molecular mechanisms that promote the angiotropic phenotype of melanoma. GSEA of DEGs between C918 and OCM-1A suggested 20 overexpressed genes. Among the 20 genes, TFPI2 was further analyzed because it overlapped with cell line DEGs and DEGs of TCGA-UM metastasis (+) *vs.* metastasis (−). TFPI2, a serine proteinase inhibitor and an ECM protein, is involved in the inhibition of ECM degradation and is associated with disease progression (43). However, its role in cancer has remained paradoxical. Previous work suggested that TFPI2 could cause decreased invasiveness and/or proliferation of gliomas, glioblastoma, breast cancer (44, 45, 56), and melanomas (46, 57). Transcriptional silencing of TFPI2 by promoter methylation has been reported in a variety of tumors including melanoma (58). Methylated TFPI2 DNA in serum from melanoma patients was more strongly associated with metastatic disease than primary disease (29). Here, we show that high levels of TFPI2 were statistically correlated with poor outcomes in UM individuals but better outcomes in CM individuals, which may be due to the few overlapping etiologies, mutation profiles, genetic signatures, and clinical behaviors of these two types of melanoma (59). On the one hand, TFPI2 can inhibit plasmin-matrix metalloproteinase activation in the invasion and metastasis of various malignancies. On the other hand, TFPI2 also induced invasion of hepatocellular carcinoma cells and contributed to vasculogenic mimicry plasticity of melanoma (31, 33). Intriguingly, TFPI2 shares a common protease target, plasmin activity, with SerpinB2, and the latter promotes the survival of brain vascular coopting cancer cells (12). Therefore, whether TFPI2 also functions in angiotropism in various malignancies and by which signaling pathways it plays a role are not clear, and more research is needed.

### Opposite Roles of TFPI2 in Survival of UM and CM

This study described opposite roles of TFPI2 in the survival of UM and CM patients. TFPI2-high predicted a poor OS in UM but no difference in long-term survival (OS and 5-year), while a contrasting better 2-year survival in CM. Despite being derived from the neural crest of common origin, UM and CM have few overlapping genetic signatures. To understand the potential mechanisms of TFPI2 function in UM and CM, coexpression analysis revealed differentially expressed genes and molecular networks that were correlated with TFPI2 expression levels in UM and CM. Specifically, in UM, TFPI2 coexpression genes are mainly associated with pathways of human papillomavirus infection and calcium signaling, while in CM, TFPI2 coexpression genes are mainly associated with cytokine–cytokine receptor interaction and TNF signaling pathways.

Additionally, this situation in which the same molecule can be expressed and be related to a better or worse overall survival in UM and CM also occurred with the deubiquitinase BRCA1-Associated Protein 1 (BAP1). Loss of BAP1 is common in UM and is associated with a worse prognosis; it is rare in CM and is associated with better OS in CM (60). Because an increased frequency of loss-of-function mutations in BAP1 in UM that become metastatic and an association between BAP1 mutation and worse prognosis/survival in UM patients were observed, we sought to examine the correlation of TFPI2 and BAP1 in mRNA expression levels from TCGA-UM and TCGA-CM data. The results showed that the mRNA of TFPI2 was negatively correlated with that of BAP1 in both UM (*P* = 0.0002, Pearson r = −0.41) and CM (*P* = 0.004, Pearson r = −0.13), but with a stronger correlation coefficient in UM (**Supplementary Figure 9**). Combined with the results that higher levels TFPI2 in metastasis (+) UM and no difference in metastasis (+) and (−) CM groups, stronger negative correlation between TFPI2 and BAP1 may indirectly and partially explain the opposite roles of TFPI2 in survival of UM and CM.

### Conclusion

We report an *in vivo* angiotropic model for direct observation of vessel–cancer cell interactions. Data acquired through bioinformatics analyses supply molecular information related to angiotropism formation, and TFPI2 could serve as a positive regulator in the angiotropic phenotype of melanoma

## Data Availability Statement

The datasets presented in this study can be found in online repositories. The names of the repository/repositories and accession number(s) can be found in the article/**Supplementary Material**.

## Ethics Statement

The studies involving human participants were reviewed and approved by Ethics Committee of Tianjin Medical University. Written informed consent to participate in this study was provided by the participants’ legal guardian/next of kin. The animal study was reviewed and approved by the Institutional Animal Care and Use Committee of Tianjin Medical University.

## Author Contributions

JM: Data curation, formal analysis, project administration, writing—original draft, and writing—review and editing. XZ: Project administration. WW: Software. NZ: Investigation. XD: Methodology. YZ: Investigation. RC: Resources. BS: Writing—review and editing. All authors contributed to the article and approved the submitted version.

## Funding

This study was supported by grants from the National Natural Science Foundation of China (No. 82002901).

## Conflict of Interest

The authors declare that the research was conducted in the absence of any commercial or financial relationships that could be construed as a potential conflict of interest.

## Supplementary Material

Supplementary Figure 1 Stronger interaction of C918 cells with the endothelium **(A)** A representative IF image of coculture of HUVEC-GFP cells with melanoma-mCherry cells, showing that C918 (red) cells contributed to capillary-like structure formation (green) and were elongated along the tubules. In contrast, OCM-1A (red) cells were scattered among the HUVEC frameworks (green). Scale bar 100μm. **(B)** Elongated cells adherent to HUVECs are shown, as measured by fluorescence microscopy (10 fields from three independent experiments). **P* < 0.05. **(C)** Aortas were isolated from 8- to 10-week-old mice, sectioned into 1- to 1.5-mm-thick rings and embedded in Matrigel for 3 days in Opti-MEM medium in 24 plates. For coculture experiments, GFP-expressing melanoma cells suspended in Matrigel were inoculated 2-3 mm away from the aortic ring. Phase contrast and fluorescence microscopy were used to observe endothelial sprouts and melanoma cells. Optical images of coculture of rat aortic rings and melanoma cells (upper panel). The fluorescence microscopy images show that C918-GFP cells appeared as a pericyte-like shape along endothelial cells sprouting from aortic rings. The morphology of OCM-1A-GFP cells grown on aortic rings did not have a tendency to become elongated (lower panel). Scale bar 100 μm.

Supplementary Figure 2 Representative scan of a coxenograft tissue section (C918-GFP+OCM-1A-con) showing the distribution of C918-GFP cells.

Supplementary Figure 3 Gene set enrichment analysis and leading-edge analysis **(A)** GSEA of underlying biological processes involved in angiotropic phenotypes of highly and poorly invasive cell lines. The top 7 GO signals significantly and positively associated with the C918 cell line are shown. **(B)** Representation of 20 genes (vertical clustering) present in the leading edges of the enriched gene sets (horizontal clustering) after GSEA. The cut-off of the false discovery rate (FDR) was set to 0.05 for the significant GO terms.

Supplementary Figure 4 Gene Ontology and KEGG pathway enrichment analysis of DEGs of the cell lines on STRING**(A)** Genes clustered according to molecular function and biological process. **(B)** KEGG pathway enrichment analysis.

Supplementary Figure 5 Expression of TFPI2 by C918 and OCM-1A cell lines Western blot analysis of protein expression of TFPI-2. The upper panel shows the comparison of supernatant from melanoma cells as indicated; the lower panel shows the comparison of whole cell lysate from melanoma cells as indicated.

Supplementary Figure 6 Comparison of the expression and methylation levels of TFPI2 The dot plots show mRNA expression **(A)** and methylation levels **(B)** of TFPI2 in TCGA-UM and TCGA-CM datasets. The histogram shows the proportions of TFPI2 methylation in the above datasets **(C)**.

Supplementary Figure 7 Functional analysis of coexpressed genes of TFPI2 in melanoma Functional analysis of coexpressed genes of TFPI2 in TCGA-UM **(A)** and TCGA-CM **(B)** by KEGG pathway enrichment analysis. **(C)** The Venn diagram shows the overlapping genes among coexpressed genes of TFPI2 in TCGA-UM and TCGA-CM, which were further investigated by KEGG pathway enrichment analysis **(D)**.

Supplementary Figure 8 Prognostic significance of angiotropism in melanoma Kaplan–Meier estimates showed that there were no significant differences in 5-year or overall survival time between angiotropism-present and angiotropism-absent patients in TMU-CM cohort.

Supplementary Figure 9 Negative correlation between expression of TFPI2 and BAP1 Stronger correlation of TFPI2 with BAP1 mRNA expression levels from TCGA-UM data than TCGA-CM.
