# Supplementary figures and images for: TFPI2 Promotes Perivascular Migration in an Angiotropism Model of Melanoma
Source: Front Oncol. 2021 Jun 24;11:662434. doi: 10.3389/fonc.2021.662434 (PMC8264799; doi:10.3389/fonc.2021.662434)

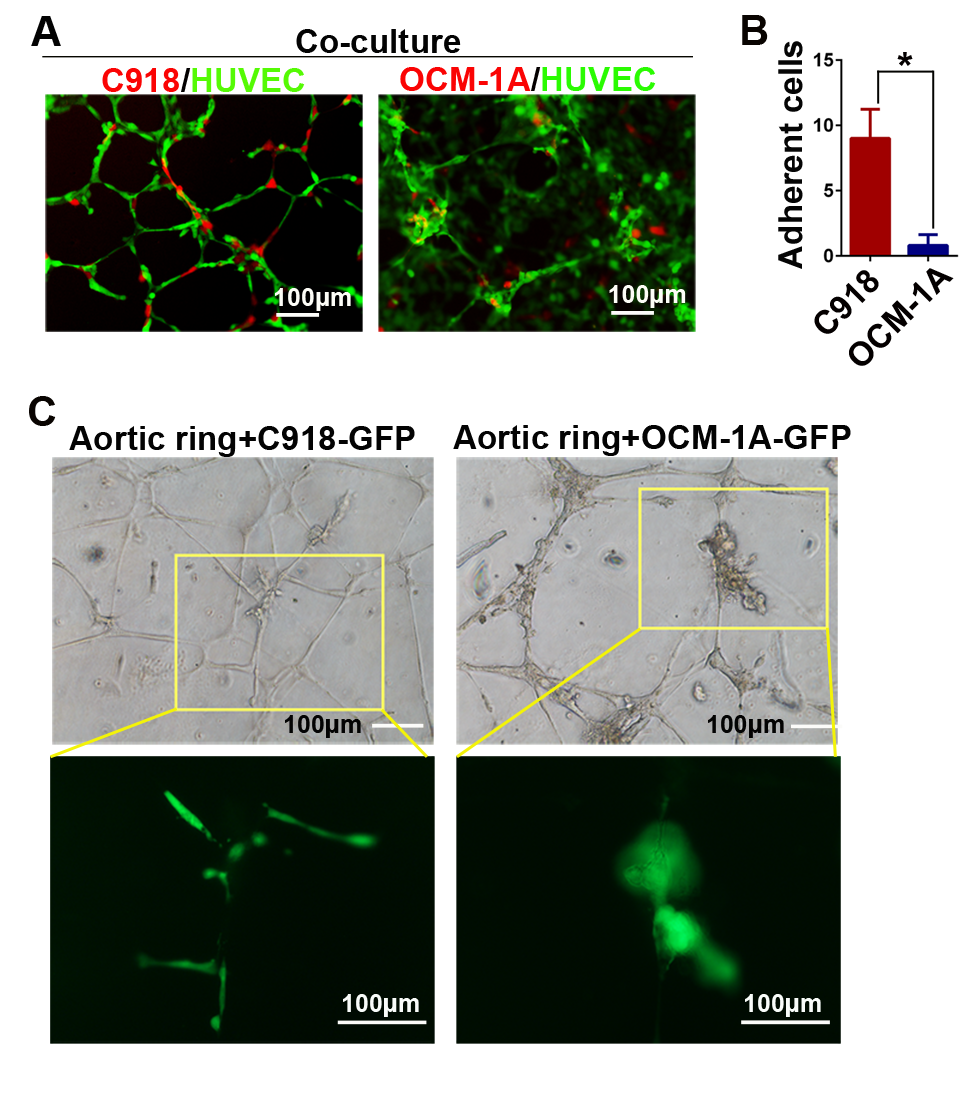

Supplement: Supplementary Figure 1 — Stronger interaction of C918 cells with the endothelium (A) A representative IF image of coculture of HUVEC-GFP cells with melanoma-mCherry cells, showing that C918 (red) cells contributed to capillary-like structure formation (green) and were elongated along the tubules. In contrast, OCM-1A (red) cells were scattered among the HUVEC frameworks (green). Scale bar 100μm. (B) Elongated cells adherent to HUVECs are shown, as measured by fluorescence microscopy (10 fields from three independent experiments). *P < 0.05. (C) Aortas were isolated from 8- to 10-week-old mice, sectioned into 1- to 1.5-mm-thick rings and embedded in Matrigel for 3 days in Opti-MEM medium in 24 plates. For coculture experiments, GFP-expressing melanoma cells suspended in Matrigel were inoculated 2-3 mm away from the aortic ring. Phase contrast and fluorescence microscopy were used to observe endothelial sprouts and melanoma cells. Optical images of coculture of rat aortic rings and melanoma cells (upper panel). The fluorescence microscopy images show that C918-GFP cells appeared as a pericyte-like shape along endothelial cells sprouting from aortic rings. The morphology of OCM-1A-GFP cells grown on aortic rings did not have a tendency to become elongated (lower panel). Scale bar 100 μm. [file Image_1.tif]

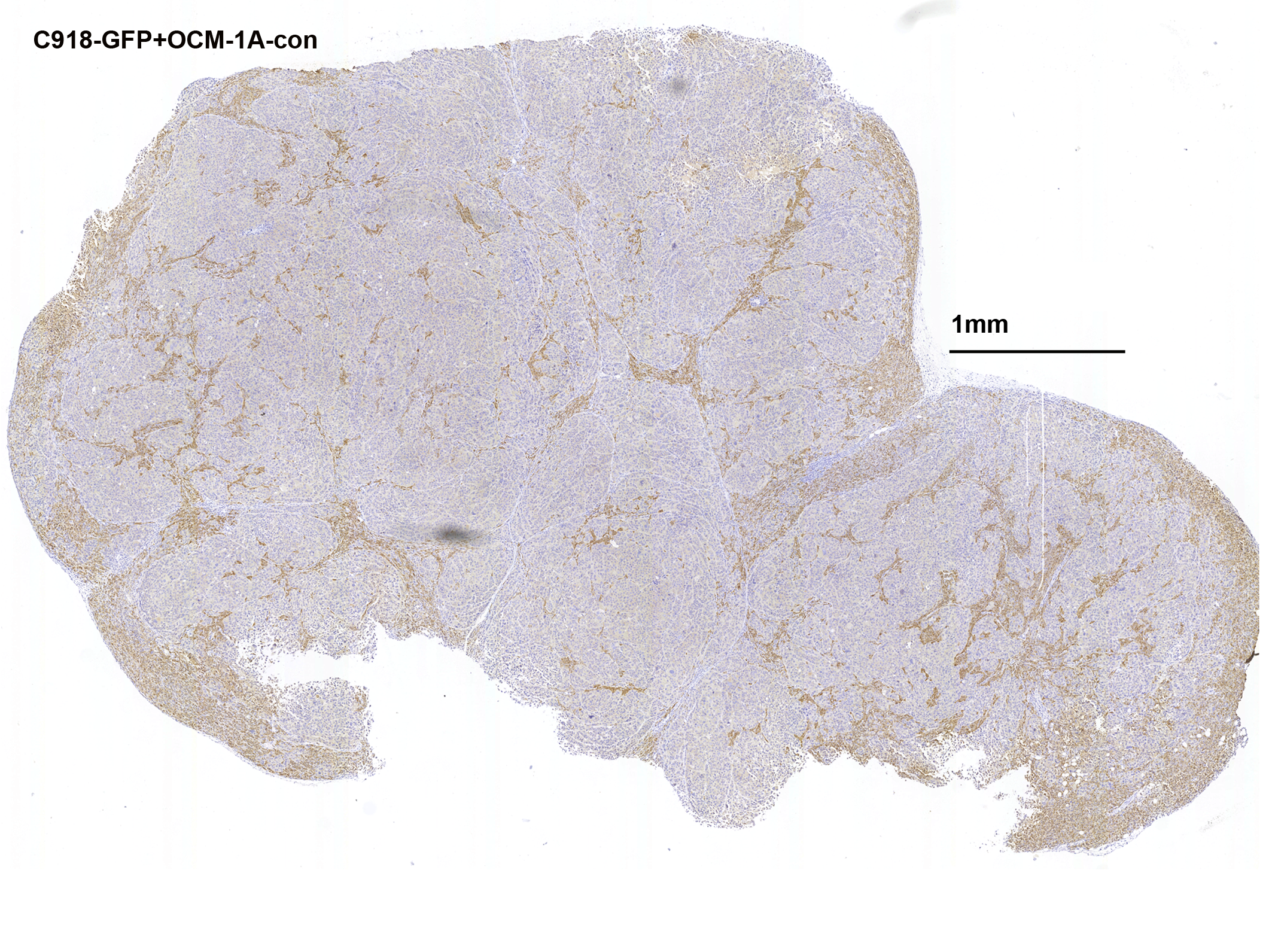

Supplement: Supplementary Figure 2 — Representative scan of a coxenograft tissue section (C918-GFP+OCM-1A-con) showing the distribution of C918-GFP cells. [file Image_2.tif]

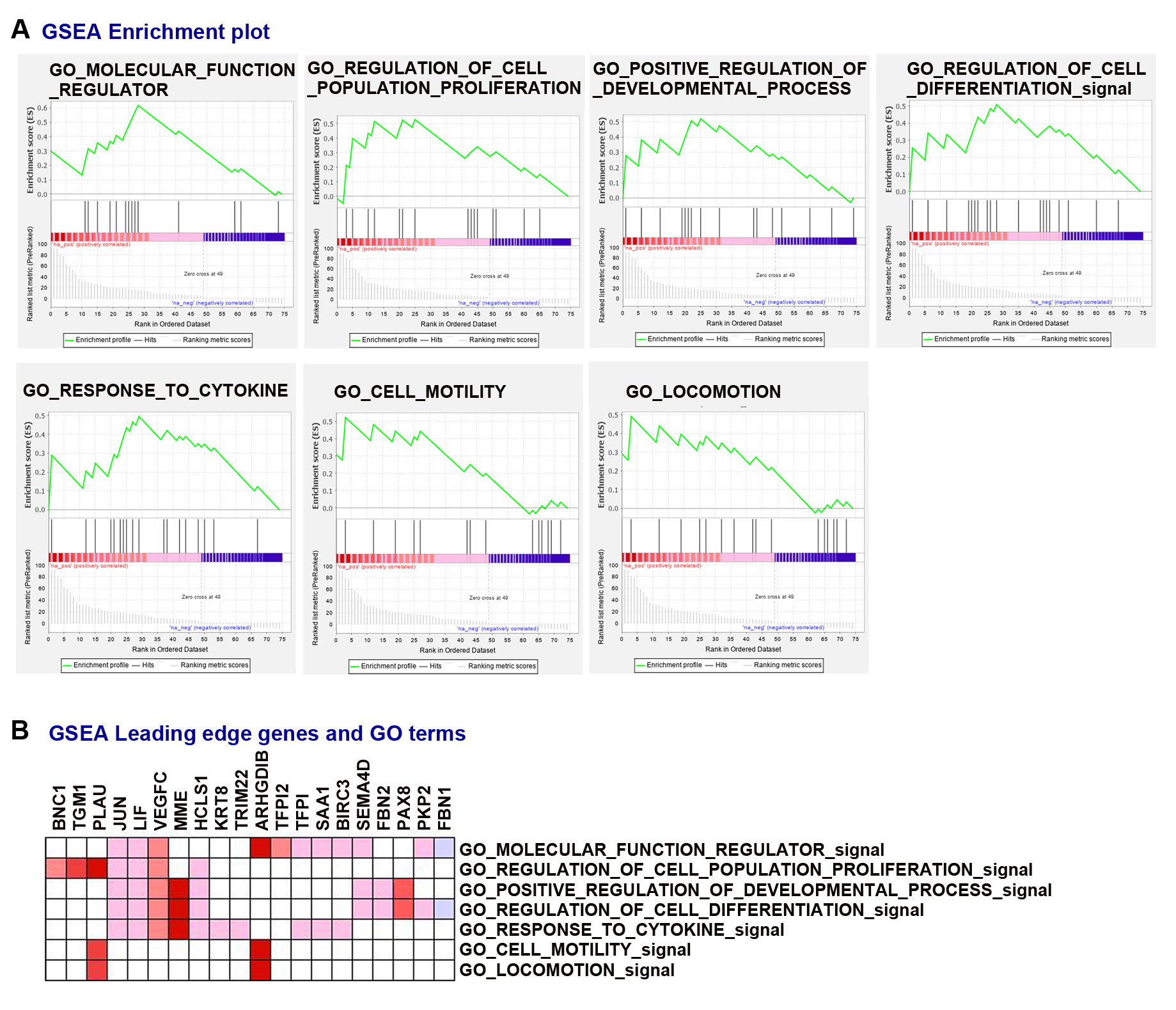

Supplement: Supplementary Figure 3 — Gene set enrichment analysis and leading-edge analysis (A) GSEA of underlying biological processes involved in angiotropic phenotypes of highly and poorly invasive cell lines. The top 7 GO signals significantly and positively associated with the C918 cell line are shown. (B) Representation of 20 genes (vertical clustering) present in the leading edges of the enriched gene sets (horizontal clustering) after GSEA. The cut-off of the false discovery rate (FDR) was set to 0.05 for the significant GO terms. [file Image_3.tif]

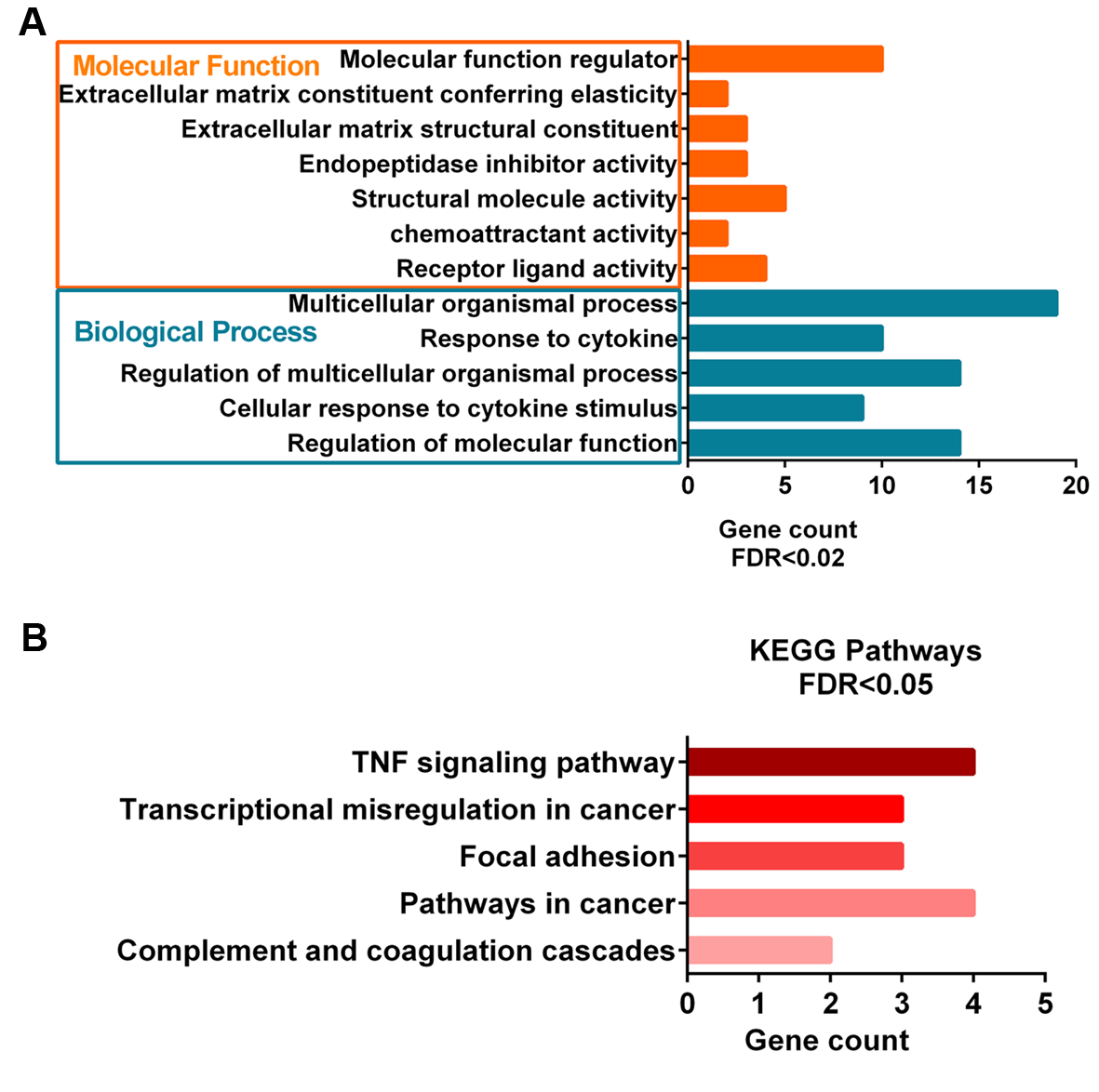

Supplement: Supplementary Figure 4 — Gene Ontology and KEGG pathway enrichment analysis of DEGs of the cell lines on STRING(A) Genes clustered according to molecular function and biological process. (B) KEGG pathway enrichment analysis. [file Image_4.tif]

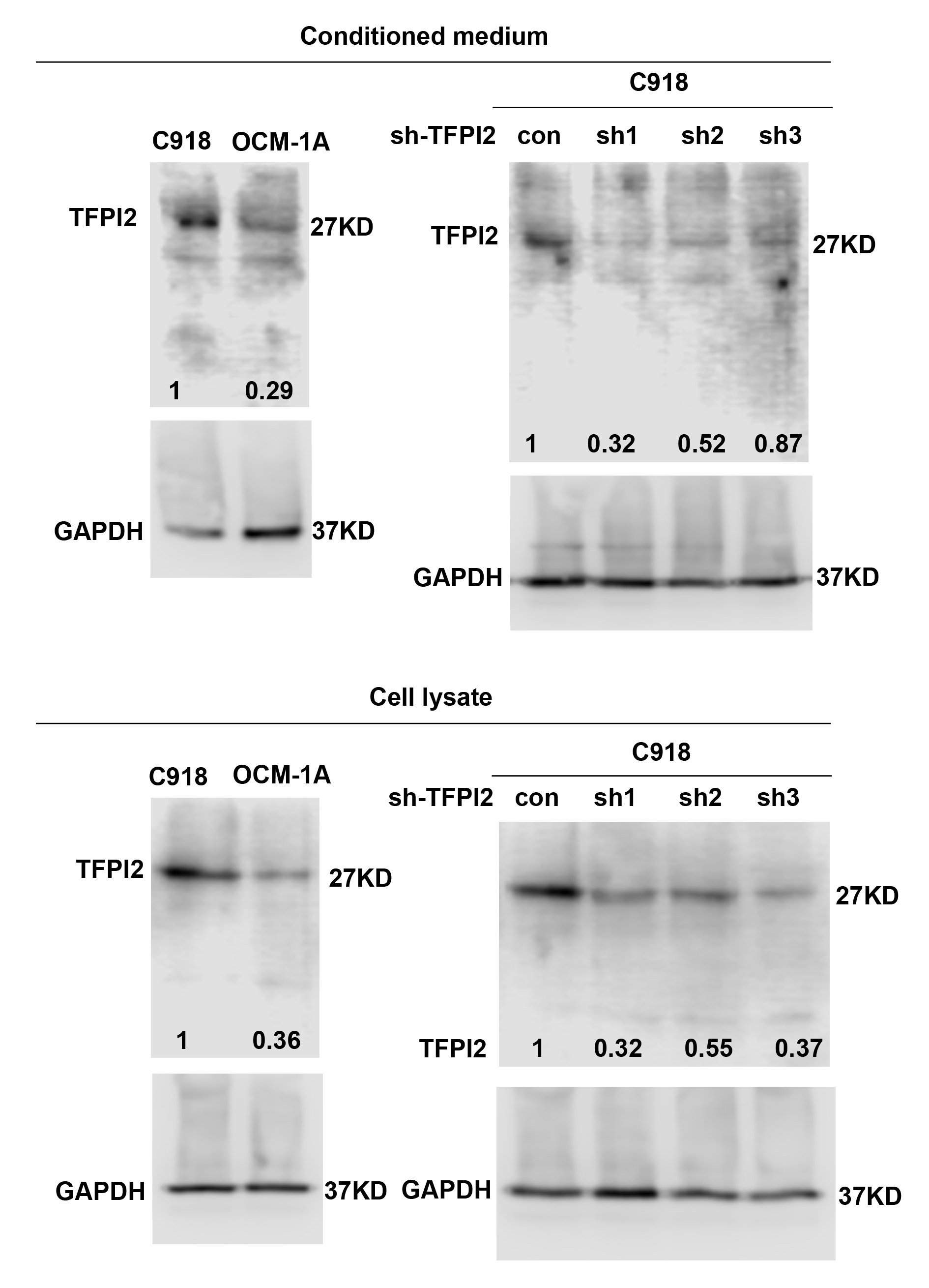

Supplement: Supplementary Figure 5 — Expression of TFPI2 by C918 and OCM-1A cell lines Western blot analysis of protein expression of TFPI-2. The upper panel shows the comparison of supernatant from melanoma cells as indicated; the lower panel shows the comparison of whole cell lysate from melanoma cells as indicated. [file Image_5.tif]

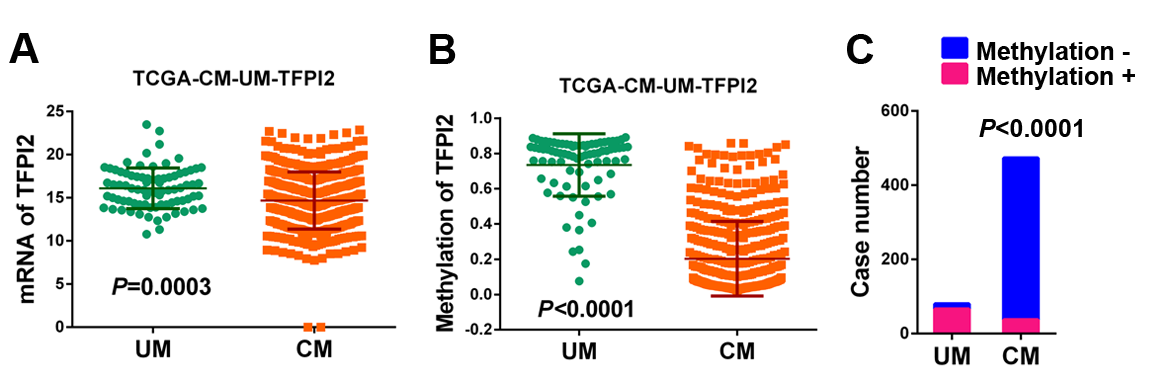

Supplement: Supplementary Figure 6 — Comparison of the expression and methylation levels of TFPI2 The dot plots show mRNA expression (A) and methylation levels (B) of TFPI2 in TCGA-UM and TCGA-CM datasets. The histogram shows the proportions of TFPI2 methylation in the above datasets (C). [file Image_6.tif]

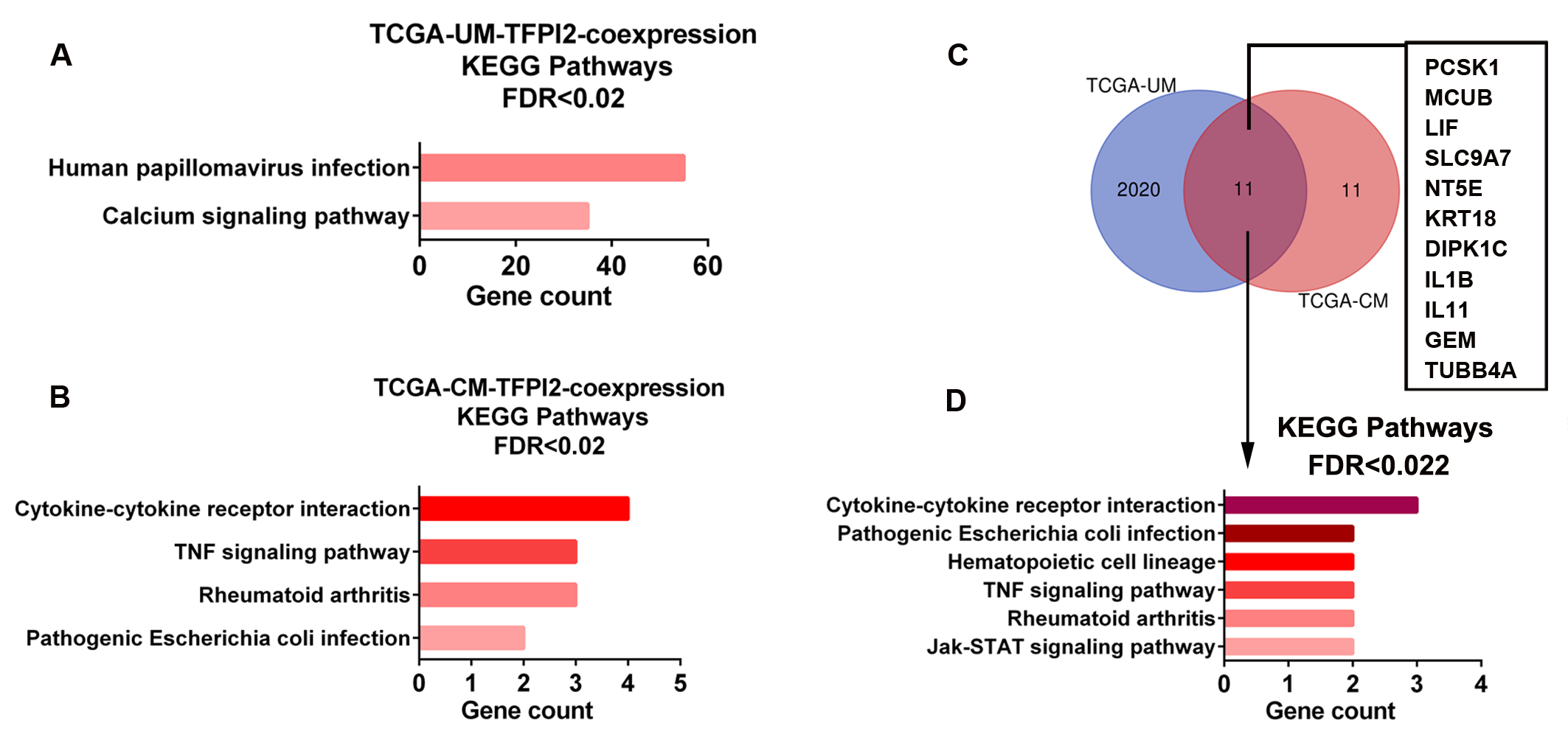

Supplement: Supplementary Figure 7 — Functional analysis of coexpressed genes of TFPI2 in melanoma Functional analysis of coexpressed genes of TFPI2 in TCGA-UM (A) and TCGA-CM (B) by KEGG pathway enrichment analysis. (C) The Venn diagram shows the overlapping genes among coexpressed genes of TFPI2 in TCGA-UM and TCGA-CM, which were further investigated by KEGG pathway enrichment analysis (D). [file Image_7.tif]

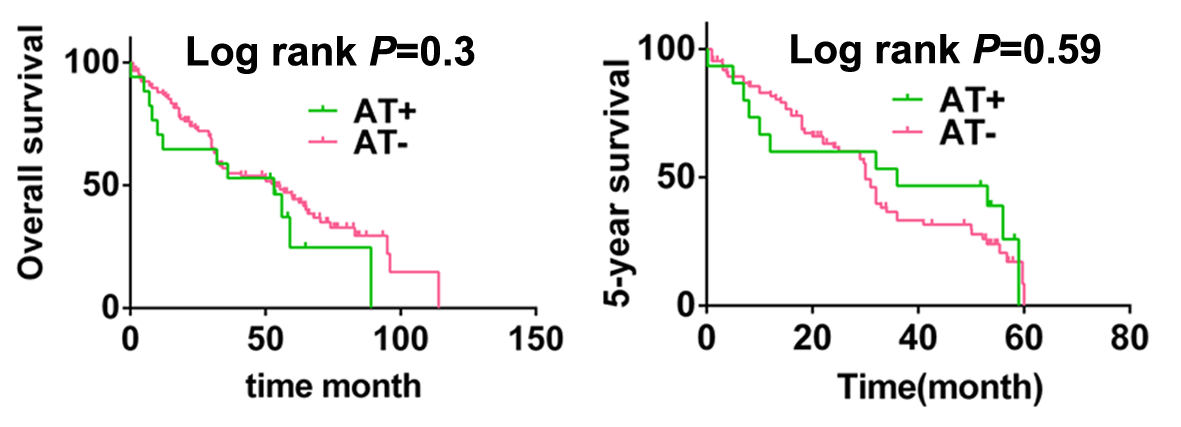

Supplement: Supplementary Figure 8 — Prognostic significance of angiotropism in melanoma Kaplan–Meier estimates showed that there were no significant differences in 5-year or overall survival time between angiotropism-present and angiotropism-absent patients in TMU-CM cohort. [file Image_8.tif]

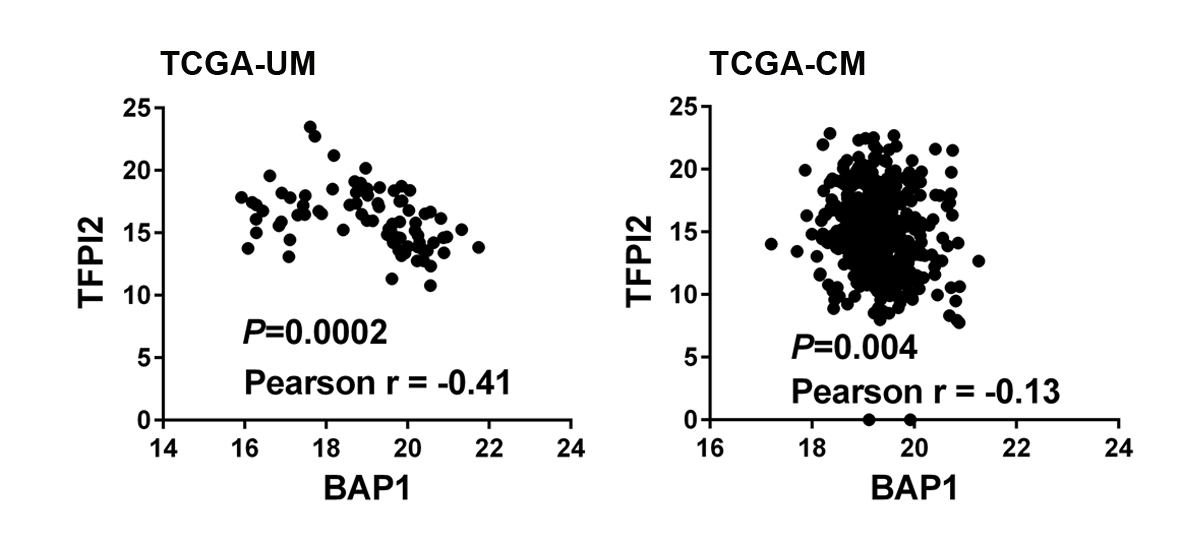

Supplement: Supplementary Figure 9 — Negative correlation between expression of TFPI2 and BAP1 Stronger correlation of TFPI2 with BAP1 mRNA expression levels from TCGA-UM data than TCGA-CM. [file Image_9.tif]
