# Supplementary material for: TFPI2 Promotes Perivascular Migration in an Angiotropism Model of Melanoma
Source: Front Oncol. 2021 Jun 24;11:662434. doi: 10.3389/fonc.2021.662434 (PMC8264799; doi:10.3389/fonc.2021.662434)
Supplement: Supplementary file 10 [file DataSheet_1.docx]

Article title:

TFPI2 promotes perivascular migration in an angiotropism model of melanoma

Journal name:

Frontiers in oncology

Author names:

Jing Mo, Xiulan Zhao, Wei Wang, Nan Zhao, Xueyi Dong, Yanhui Zhang,

Runfen Cheng, Baocun Sun*

*Corresponding author:
 Baocun Sun

E-mail: baocunsun@gmail.com

**Supplementary Table.1 DEGs of highly and poorly invasive melanoma cell lines**

| **up regulated genes:** | **Ratio** |  | **up regulated genes:** | **Ratio** |  | **down regulated genes:** | **Ratio** |
| --- | --- | --- | --- | --- | --- | --- | --- |
| CALLA | 100 |  | JUN | 16 |  | SIAT1 | -4.7 |
| TIE | 100 |  | STAF50 | 15 |  | IRS1 | -4.8 |
| ARHGDIB | 100 |  | FBN1 | 15 |  | TYR | -4.9 |
| PLAU | 80 |  | IGFBP1 | 14 |  | RARB | -4.9 |
| ECK | 77 |  | S100A1 | 13 |  | ABCC2 | -5 |
| TGM1 | 61 |  | UPAR | 11 |  | MLANA | -5.1 |
| PAX8 | 58 |  | NNMT | 10 |  | SIAT8A | -5.1 |
| TM4SF1 | 52 |  | SMTN | 8.5 |  | HLA-DMA | -5.2 |
| TWIK-1 | 45 |  | P37NB | 8.4 |  | LUM | -5.3 |
| CTGF | 35 |  | ALCAM | 8.3 |  | GSTM4 | -5.5 |
| BNC | 31 |  | CA1 | 8.3 |  | APOC2 | -6 |
| TFPI2 | 31 |  | IL15RA | 7.7 |  | S100B | -6.4 |
| VEGFC | 30 |  | ETS2 | 7.3 |  | PLP1 | -6.5 |
| CPS1 | 28 |  | FLI1 | 6.9 |  | S100P | -6.5 |
| RIG | 26 |  | ETS1 | 6.8 |  | A2M | -6.5 |
| KRT7 | 25 |  | COL6A3 | 6.8 |  | CYP27A1 | -6.7 |
| API2 | 25 |  | DDR2 | 6.3 |  | APOD | -6.9 |
| TCEB3 | 22 |  | NFKBIA | 5.6 |  | ABCC1 | -7 |
| LTBP2 | 21 |  | MSX1 | 5.4 |  | TF | -7.6 |
| SEMA4D | 20 |  | LSP1 | 4.8 |  | CTH | -7.6 |
| LIF | 19 |  | DTR | 4.7 |  | STHM | -7.7 |
| HCLS1 | 19 |  | COL1A1 | 4.6 |  | CHL1 | -7.7 |
| TFPI | 18 |  |  | |  | ESTs | -7.8 |
| KRT8 | 18 |  |  |  |  | MYLK | -7.9 |
| FBN2 | 18 |  |  |  |  | NAIP | -8.4 |
| PKP2 | 16 |  |  |  |  | GPM6B | -10 |
| SAA1 | 16 |  |  |  |  |  |  |

**Supplementary Table. 2 Correlations between the 20 key genes obtained from GSEA and overall survival of TCGA-UM patients by univariate and multivariable Cox proportional hazards analysis**

|  | **Univariate analysis** | | | |  | **Multivariate analysis** |
| --- | --- | --- | --- | --- | --- | --- |
| **Variables** | ***P* value** | **Exp(B)** | **CI Lower** | **CI Upper** |  | ***P* value** |
| **BNC1** | 0.099 | 1.196 | 0.967 | 1.478 |  | 0.35 |
| **TGM1** | 0.752 | 0.965 | 0.773 | 1.204 |  |  |
| **PLAU** | 0.412 | 1.247 | 0.736 | 2.114 |  |  |
| **JUN** | 0.052 | 1.952 | 0.995 | 3.833 |  | 0.129 |
| **LIF** | 0.024* | 1.5 | 1.054 | 2.132 |  | 0.39 |
| **VEGFC** | 0.037* | 1.52 | 1.025 | 2.255 |  | 0.11 |
| **MME** | 0.061 | 0.821 | 0.667 | 1.009 |  | 0.73 |
| **HCLS1** | 0.998 | 0.999 | 0.64 | 1.56 |  |  |
| **KRT8** | 0.088 | 0.722 | 0.497 | 1.05 |  | 0.9 |
| **TRIM22** | 0.787 | 1.045 | 0.758 | 1.44 |  |  |
| **ARHGDIB** | 0.322 | 0.701 | 0.346 | 1.417 |  |  |
| **SAA1** | 0.254 | 1.091 | 0.94 | 1.266 |  |  |
| **BIRC3** | 0.083 | 1.276 | 0.969 | 1.682 |  | 0.8 |
| **FBN1** | 0.629 | 1.143 | 0.664 | 1.968 |  |  |
| **SEMA4D** | 0.89 | 1.082 | 0.355 | 3.303 |  |  |
| **FBN2** | 0.898 | 1.032 | 0.641 | 1.661 |  |  |
| **PAX8** | 0.189 | 1.143 | 0.936 | 1.396 |  |  |
| **PKP2** | 0.017 | 0.623 | 0.422 | 0.92 |  | 0.13 |
| **TFPI** | 0.431 | 0.871 | 0.618 | 1.228 |  |  |
| **TFPI2** | 0.003** | 1.355 | 1.108 | 1.658 |  | 0.102 |

CI, Confidence interval. **P*<0.05; ***P*<0.005

**Supplementary Table. 3 Correlations between the 20 key genes obtained from GSEA and overall survival of TCGA-CM patients by univariate and multivariable Cox proportional hazards analysis**

|  | **Univariate analysis** | | | |  | **Multivariate analysis** |
| --- | --- | --- | --- | --- | --- | --- |
| **Variables** | ***P* value** | **Exp(B)** | **CI Lower** | **CI Upper** |  | ***P* value** |
| **BNC1** | 0.287 | 1.02 | 0.98 | 1.07 |  |  |
| **TGM1** | 0.136 | 1.07 | 0.98 | 1.19 |  |  |
| **PLAU** | 0.78 | 0.98 | 0.88 | 1.09 |  |  |
| **JUN** | 0.99 | 0.99 | 0.86 | 1.16 |  |  |
| **LIF** | 0.004** | 0.91 | 0.86 | 0.97 |  | 0.19 |
| **VEGFC** | 0.63 | 1.03 | 0.89 | 1.19 |  |  |
| **MME** | 0.07 | 1.05 | 0.99 | 1.10 |  |  |
| **HCLS1** | 0.005* | 0.86 | 0.78 | 0.96 |  |  |
| **KRT8** | 0.27 | 0.96 | 0.90 | 1.03 |  |  |
| **TRIM22** | 0.00002** | 0.80 | 0.73 | 0.89 |  | 0.012* |
| **ARHGDIB** | 0.047* | 0.89 | 0.79 | 0.99 |  | 0.43 |
| **SAA1** | 0.71 | 0.99 | 0.96 | 1.03 |  |  |
| **BIRC3** | 0.002** | 0.88 | 0.81 | 0.95 |  | 0.8 |
| **FBN1** | 0.77 | 1.02 | 0.91 | 1.13 |  |  |
| **SEMA4D** | 0.0001** | 0.78 | 0.69 | 0.88 |  | 0.044* |
| **FBN2** | 0.28 | 1.05 | 0.96 | 1.14 |  |  |
| **PAX8** | 0.28 | 0.96 | 0.90 | 1.03 |  |  |
| **PKP2** | 0.69 | 1.01 | 0.95 | 1.07 |  |  |
| **TFPI** | 0.37 | 1.05 | 0.94 | 1.18 |  |  |
| **TFPI2** | 0.55 | 0.99 | 0.94 | 1.03 |  |  |

CI, Confidence interval. **P*<0.05; ***P*<0.005

**Supplementary Table. 4 Correlations between the clinicopathological parameters and overall survival of TCGA-UM patients by univariate and multivariable Cox proportional hazards analysis**

| **Variable** | **Univariate analysis** | |  | **Multivariate analysis** | |
| --- | --- | --- | --- | --- | --- |
|  | ***P* value** | **Hazard ratio (95% CI)** |  | ***P* value** | **Hazard ratio (95% CI)** |
| **Age** | 0.02* | 1.1（1.0-1.1） |  | 0.001** | 1.108(1.04-1.18) |
| **Gender** | 0.35 | 1.7（0.5-5.7） |  |  |  |
| **Thickness** | 0.57 | 1.1（0.9-1.3） |  |  |  |
| **Diameter** | 0.07 | 1.2（1.0-1.5） |  | 0.8 |  |
| **Mitotic rate** | 0.9 | 1.0（0. 9-1.1） |  |  |  |
| **Stage** | 0.008* | 5.9（1.6-21.7） |  | 0.021* | 4.273(1.24-14.71) |
| **Metastasis** | 0.008* | 7.7（1.7-35.2） |  | 0.01* | 8.366(1.66-42.07) |
| **TFPI2** | 0.003** | 1.4（1.1-1.7） |  | 0.10 |  |

CI, Confidence interval. **P*<0.05; ***P*<0.005

**Supplementary Table. 5 Correlations between TFP2 and clinicopathological parameters of TCGA-UM**

| **Variables** | | **n** | **TFPI2** | |
| --- | --- | --- | --- | --- |
|  |  |  | ***P* value** | **Pearson r** |
| **Median age, years (range)** | 60(22-86) | 80 | 0.1019 (Pearson) | 0.1842 |
| **Sex, female/male** | 35/45 | 80 | 0.5605 (t test) |  |
| **Thickness, mm (range)** | 10.4(4-16) | 80 | 0.1131 (Pearson) | 0.1785 |
| **Diameter, mm (range)** | 16.9(7.79-25) | 79 | 0.0273 (Pearson)* | 0.2484 |
| **Median mitotic count, /mm^2^ (range )** | 7(0-40) | 53 | 0.0161(Pearson)* | 0.3292 |
| **Stage , n** |  | 80 | 0.0045 (t test)** |  |
| **I-II, n (%)** | 36(45) |  |  |  |
| **III- IV, n (%)** | 44(55) |  |  |  |
| **Metastasis, n (%)** | 27(33.8) | 80 | 0.0019 (t test) ** |  |

CI, Confidence interval. **P*<0.05; ***P*<0.005

**Supplementary Table. 6 Correlations between TFP2 and clinicopathological parameters of TCGA-CM**

| **Variables** | | **n** | **TFPI2** | |
| --- | --- | --- | --- | --- |
|  |  |  | ***P* value** | **Pearson r** |
| **Median age, years (range)** | 58(15-90) | 460 | 0.0064 (Pearson)* | -0.1268 |
| **Sex, female/male** | 179/289 | 468 | 0.7855 (t test) |  |
| **Clark** |  | 319 | 0.0746 (t test) |  |
| **I- III, n (%)** | 100(31.3) |  |  |  |
| **IV-V, n (%)** | 219(68.7) |  |  |  |
| **Median Breslow thickness, mm (range)** | 3(0-75) | 357 | 0.6239 (Pearson) | -0.026 |
| **Median mitotic count, /mm^2^ (range )** | 5(0-40) | 170 | 0.1367 (Pearson) | -0.1146 |
| **Ulceration present,** **n (%)** | 165(53.1) | 311 | 0.214(t test) |  |
| **Stage , n** |  | 430 | 0.4085(t test) |  |
| **I-II, n (%)** | 237(55.1) |  |  |  |
| **III- IV, n (%)** | 193(44.9) |  |  |  |
| **Metastasis, n (%)** | 203(43.4) | 468 | 0.6826(t test) |  |

**P*<0.05

**Supplementary Table. 7 Correlations between the clinicopathological parameters and overall survival of TCGA-CM patients by univariate and multivariable Cox proportional hazards analysis**

| **Variable** |  | **Univariate analysis** | | **Multivariate analysis** | |
| --- | --- | --- | --- | --- | --- |
|  |  | ***P* value** | **Hazard ratio (95% CI)** | ***P* value** | **Hazard ratio (95% CI)** |
| **Age** |  | 0.001** | 2.0（1.3-3.0） | 0.031* | 1.021 (1.002–1.041) |
| **Gender** |  | 0.31 | 1.2（0.8-1.7） |  |  |
| **Clark** |  | 0.001** | 2.0（1.3-3.0） |  |  |
| **Breslow thickness** |  | <0.001** | 2.5（1.7-3.6） | <0.001** | 1.049 (1.022–1.076) |
| **Mitotic rate** |  | 0.015* | 2.1（1.2-3.8） |  |  |
| **Ulceration** |  | 0.001** | 2.1（1.4-3.1） |  |  |
| **Stage** |  | 0.001** | 1.9（1.3-2.7） | 0.002** | 1.792(1.241-2.586) |
| **Metastasis** |  | 0.016* | 1.5（1.1-2.1） | 0.006* | 3.906(1.486-10.204) |
| **TFPI2** |  | 0.124 | 1.28（0.93-1.77） | 0.78 |  |

CI, Confidence interval. **P*<0.05; ***P*<0.005

**Supplementary Table. 8 Correlations between TFPI2 and clinicopathological parameters of 135 melanoma patients in TMU-CM cohort**

| **Variables** | **Number** | **TFPI2** | |  | |
| --- | --- | --- | --- | --- | --- |
|  |  | **Low** | **High** | ***χ2*** | ***P* value** |
| **Age (years)** |  |  |  | 0.48 | 0.49 |
| **＜60** | 58 | 30 | 28 |  |  |
| **≥60** | 55 | 32 | 23 |  |  |
| **Sex,** |  |  |  | 0.21 | 0.65 |
| **Female** | 38 | 22 | 16 |  |  |
| **Male** | 75 | 40 | 35 |  |  |
| **Ulceration** |  |  |  | 0.47 | 0.49 |
| **Abscent** | 47 | 24 | 23 |  |  |
| **Present** | 66 | 38 | 28 |  |  |
| **Stage** |  |  |  | 2.88 | 0.09 |
| **I-II** | 23 | 9 | 14 |  |  |
| **III- IV** | 90 | 53 | 37 |  |  |
| **LN metastasis** |  |  |  | 3.19 | 0.07 |
| **Abscent** | 56 | 26 | 30 |  |  |
| **Present** | 57 | 36 | 21 |  |  |
| **Metastasis** |  |  |  | 0.47 | 0.49 |
| **Abscent** | 66 | 38 | 28 |  |  |
| **Present** | 47 | 24 | 23 |  |  |

**Supplementary Table. 9 Correlations between angiotropism and clinicopathological parameters of 135 melanoma patients in TMU-CM cohort**

| **Variables** | **Number** | **Angiotropism** | |  | |
| --- | --- | --- | --- | --- | --- |
|  |  | **Abscent** | **Present** | ***χ2*** | ***p* value** |
| **Age (years)** |  |  |  | 0.089 | 0.77 |
| **＜60** | 75 | 65 | 10 |  |  |
| **≥60** | 60 | 53 | 7 |  |  |
| **Sex,** |  |  |  | 2.53 | 0.11 |
| **Female** | 47 | 44 | 3 |  |  |
| **Male** | 88 | 74 | 14 |  |  |
| **Ulceration** |  |  |  | 0.77 | 0.38 |
| **Abscent** | 61 | 55 | 6 |  |  |
| **Present** | 74 | 63 | 11 |  |  |
| **Stage** |  |  |  | 0.17 | 0.68 |
| **I-II** | 29 | 26 | 3 |  |  |
| **III- IV** | 106 | 92 | 14 |  |  |
| **LN metastasis** |  |  |  | 0.47 | 0.52 |
| **Abscent** | 72 | 62 | 10 |  |  |
| **Present** | 63 | 56 | 7 |  |  |
| **Metastasis** |  |  |  | 0.14 | 0.71 |
| **Abscent** | 85 | 75 | 10 |  |  |
| **Present** | 50 | 43 | 7 |  |  |
